# HMGA1 drives chemoresistance in esophageal squamous cell carcinoma by suppressing ferroptosis

**DOI:** 10.1038/s41419-024-06467-2

**Published:** 2024-02-21

**Authors:** Jing-Yu Yang, Xin-Yuan Lei, Kai-Yue He, Jin-Rong Guo, Meng-Jie Liu, Jun-Qi Li, Qiu-Tong Li, Zhi-Hao Jiang, Lei Zhang, Dan-Hui Wu, Yu-Jia Li, Qian-Hui Sun, Yong-Ping Jian, Zhi-Xiang Xu

**Affiliations:** School of Life Sciences, Henan University, Kaifeng, Henan Province China

**Keywords:** Cancer epigenetics, Cancer genetics

## Abstract

Chemotherapy is a primary treatment for esophageal squamous cell carcinoma (ESCC). Resistance to chemotherapeutic drugs is an important hurdle to effective treatment. Understanding the mechanisms underlying chemotherapy resistance in ESCC is an unmet medical need to improve the survival of ESCC. Herein, we demonstrate that ferroptosis triggered by inhibiting high mobility group AT-hook 1 (HMGA1) may provide a novel opportunity to gain an effective therapeutic strategy against chemoresistance in ESCC. HMGA1 is upregulated in ESCC and works as a key driver for cisplatin (DDP) resistance in ESCC by repressing ferroptosis. Inhibition of HMGA1 enhances the sensitivity of ESCC to ferroptosis. With a transcriptome analysis and following-up assays, we demonstrated that HMGA1 upregulates the expression of solute carrier family 7 member 11 (SLC7A11), a key transporter maintaining intracellular glutathione homeostasis and inhibiting the accumulation of malondialdehyde (MDA), thereby suppressing cell ferroptosis. HMGA1 acts as a chromatin remodeling factor promoting the binding of activating transcription factor 4 (ATF4) to the promoter of SLC7A11, and hence enhancing the transcription of SLC7A11 and maintaining the redox balance. We characterized that the enhanced chemosensitivity of ESCC is primarily attributed to the increased susceptibility of ferroptosis resulting from the depletion of HMGA1. Moreover, we utilized syngeneic allograft tumor models and genetically engineered mice of HMGA1 to induce ESCC and validated that depletion of HMGA1 promotes ferroptosis and restores the sensitivity of ESCC to DDP, and hence enhances the therapeutic efficacy. Our finding uncovers a critical role of HMGA1 in the repression of ferroptosis and thus in the establishment of DDP resistance in ESCC, highlighting HMGA1-based rewiring strategies as potential approaches to overcome ESCC chemotherapy resistance.

**Figure:**
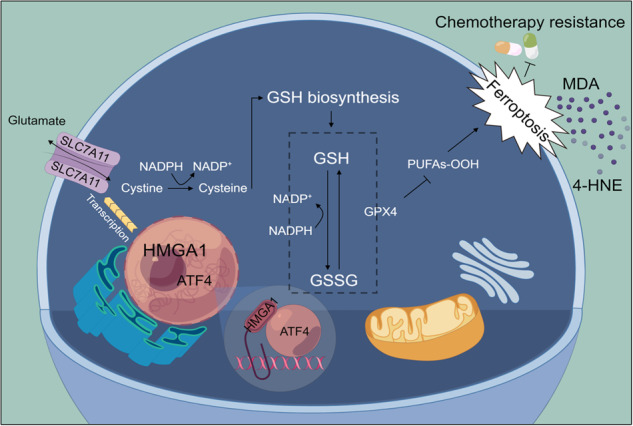
Schematic depicting that HMGA1 maintains intracellular redox homeostasis against ferroptosis by assisting ATF4 to activate SLC7A11 transcription, resulting in ESCC resistance to chemotherapy.

Schematic depicting that HMGA1 maintains intracellular redox homeostasis against ferroptosis by assisting ATF4 to activate SLC7A11 transcription, resulting in ESCC resistance to chemotherapy.

## Introduction

Esophageal cancer (EC) is a major life-threatening health problem around the world. The vast majority of ECs occur as either esophageal squamous cell carcinoma (ESCC) in the middle or upper third of the esophagus, or as esophageal adenocarcinoma (EAC) in the distal third or esophagogastric junction, with other tumor types occurring very rarely [1, 2]. ESCC accounts for 90% of ECs and is highly prevalent in the East [2, 3]. Early diagnosis of EC is difficult due to the asymptomatic characteristics of the disease in early stage. There is an obvious need for systemic treatment to improve the progression of EC, which has led to researches focusing on combined-modality therapies incorporating chemotherapy [4]. Molecular targeted therapy and immune checkpoint inhibitors have also been evaluated in clinical trials for EC recently [5, 6]. Cisplatin (DDP) was used as a treatment for ESCC and a basic agent for targeted therapy and immune checkpoint blockade in EC [7]. A broad-spectrum antitumor activity with apparent toxicities has been reported for DDP. Drug resistance is another decisive factor that affects the chemotherapeutic effects of ESCC. More than 70% of patients with locally advanced ESCC do not achieve pathological complete response (pCR) after neoadjuvant chemoradiotherapy, ultimately leading to tumor recurrence and poor prognosis [5]. These patients will require reinterventions for controlling local relapse or distant metastasis [8]. To overcome these major challenges, there is an urgent need to develop new therapies, which require further understandings to elucidate the resistance mechanisms of ESCC, whereas it is also important to investigate genes associated with development of EC and characterize their roles in the targeted therapy of EC.

High mobility group AT-hook 1 (HMGA1) is classified as an “architectural transcription factor” that plays a crucial role in chromatin structure remodeling and the regulation of transcription factor-DNA binding [9, 10]. HMGA1 has been reported to be involved in cellular transformation, and is elevated in spontaneous tumors and tumors induced by carcinogens and viral oncogenes [9]. In addition, overexpression of HMGA1 in human and murine cancer cells promotes colony formation and invasion and metastasis of cancer cells [11]. In contrast, depletion of HMGA1 not only inhibits cancer cell proliferation but also induces programmed cell death, sensitizing cancer cells to chemotherapy [12]. Additionally, HMGA1 is predicted to regulate ferroptosis-related differentially expressed genes [13]. Nevertheless, the precise regulatory role of HMGA1 in ferroptosis within EC remains unknown.

Ferroptosis, a unique modality of cell death driven by iron-dependent phospholipid peroxidation, is regulated by multiple cellular metabolic signals, including redox homeostasis, iron metabolism, mitochondrial activity and metabolism of amino acids, lipids and sugars, in addition to various disease-related signaling pathways [14, 15]. The most widely known of these is that ferroptosis is driven by lethal lipid peroxidation resulting from cellular metabolism abnormality and imbalance of redox homeostasis [16]. Ferroptosis has been reported to sensitize cancer to chemotherapy, and a synergistic effect of ferroptosis inducers and chemotherapeutic drugs can be reached and drug-resistance is reversed [17, 18]. However, it remains unknown whether targeting EC-associated genes will sensitize the tumor to chemotherapeutic drugs by inducing ferroptosis and improve the treatment outcome of EC.

Elucidation of signaling pathways governing cell death has revealed how programmed cell death is triggered in response to various physiologic stresses that cancer cells experience during the course of tumorigenesis or as a result of anticancer therapy [19, 20]. Ferroptosis is a form of oxidative cell death that is biochemically and morphologically distinct from other types of programmed cell death [15, 21]. Peroxidation disrupts the intracellular redox balance, leading to changes in MDA and GSH levels [14, 22]. Ferroptosis is driven by excessive oxidative destruction (peroxidation) of lipids in the cellular membranes [23]. Consequences of ferroptosis include the formation of various secondary lipid peroxide breakdown products, modification and oxidation of proteins, and eventual breakdown of membrane integrity. Morphologically, ferroptotic cells display small mitochondria with increased mitochondrial membrane densities, reduced or vanishing mitochondrial cristae, and rupture of the outer mitochondrial membrane [24]. In terms of the requirements for an effective execution of ferroptosis, iron ions and oxygen are sufficient to induce the programmed cell death, an oldest and evolutionary mostly conserved form of regulated cell death [25]. Notably, among the ferroptosis-inducing stresses are signaling imbalances resulting from elevated levels of oncogene signaling as well as redox balance associated with hyperproliferation [19, 20]. Thus, targeting ferroptotic signals could sensitize chemotherapeutics and reduce chemotherapy toxicity.

In the current study, we provide strong evidence demonstrating that HMGA1 enhances the recruitment and association of transcriptional factor ATF4 to the promoter of SLC7A11 and hence promotes the transcription of the gene, which functions as a suppressor of ferroptosis. Depletion of HMGA1 promotes ferroptosis and restores the sensitivity of ESCC to chemotherapy both in vitro and in vivo. Our finding uncovers a critical role of HMGA1 in the repression of ferroptosis and thus in the establishment of DDP resistance in ESCC, highlighting that targeting HMGA1 could serve as a new mechanism for alleviating ESCC chemoresistance through the induction of ferroptosis.

## Materials and methods

### Mice

Healthy C57BL/6 mice (female, 6 weeks old, weighing 18–22 g) were maintained in the SPF animal facility in Henan University and used for the establishment of animal model. For the establishment of murine subcutaneous cancer model, 5 × 10^6^/100 μl of AKR cells were inoculated into the groin of mice by subcutaneous injection. Murine weight and the tumor were monitored every other day for 15 days. From the fifth day after the subcutaneous injection, DDP (5 mg/kg) was i.p. injected for three times with one dosage each three days. Mice were randomly allocated into DDP or PBS treatment group. Single blind experiment was used for the animal therapeutic observation. Mice were maintained in 25 °C, 50% humidity, and a 12:12 day/night cycle for the entire experiment. Standard diet and purified water were offered. The animal experiment procedures were performed in accordance with the Guide of Laboratory Animal Care and Use from the Experimental Animal Association of China and were approved by the Institutional Animal Care and Use Committee (IACUC) of Henan University, China.

### Antibodies and reagents

Antibodies to HMGA1 (1:1000, sc-393213, Santa Cruz), SLC7A11 (1:1000, #12691, Cell Signaling), cleaved caspase-3 (Asp175, 1:1000, #9661, Cell Signaling), ATF4 (1:1000, #11815, Cell Signaling), DDDDK tag (1:1500, ab205606, Abcam), and β-actin (1:20000, #66009-1-Ig, Proteintech) were used for the immunoblot. HMGA1 (1:1000, sc-393213, Santa Cruz), xCT (1:200, NB300-318, NOVUS), Ki67 (1:200, ab16667, Abcam), and 4-hydroxynonenal (1:5000, MAB3249, R&D systems) were used for the immunohistochemistry. Ferrostatin-1 (HY-100579), erastin (HY-15763), staurosporine (HY-15141), z-VAD-FMK (HY-16658B), necrostatin-1 (HY-14622A), and cisplatin (HY-17394) were purchased from MCE (MCE, China).

### Cell culture

Human ESCC cell lines KYSE30 and KYSE70 were maintained in the lab. Murine ESCC cell line AKR was purchased from Otwo Biotech (HTX2545, OTWO, China). KYSE30, KYSE70, and AKR cells were cultured in RPMI1640 medium containing 10% FBS. All cells were maintained in 5% CO_2_ at 37 °C.

### Plasmid construction

To generate expression plasmids of lentiCRISPR V3-HA-Flag-HMGA1, lentiCRISPR V3-HA-Flag-SLC7A11, and lentiCRISPR V3-HA-Flag-ATF4, human HMGA1, SLC7A11, and ATF4 cDNAs were amplified with PCR and then cloned into the lentivirus vector lentiCRISPR V3-Puro with an N-terminal Flag tag with restriction enzymes *Xho*I and *EcoR*I. We employed Lipofectamine 2000 Transfection Reagent (Invitrogen, 11668030) to transduce the target plasmid and helper plasmid into 293T cells for virus production. The viruses collected after concentration were used to infect target cells, supplemented with polybrene. Puromycin was utilized to select positive cells, given that the lentiCRISPR V3 plasmid is incorporated a PuroR element, providing resistance to puromycin for the cell line following viral infection. Human and mouse-specific shRNAs targeting the HMGA1 gene, along with plasmids (LV2N(U6/Puro)-HMGA1-Homo, LV2N-shNC-Homo, LV2N(U6/Puro)-HMGA1-Mus, LV2N-shNC-Mus), as well as siRNAs targeting HMGA1 and SLC7A11, were purchased from Genepharma (Genepharma, Shanghai, China). To generate luciferase reporter vector of SLC7A11 promoter, DNA fragment of human SLC7A11 gene promoter (–2000/+500) was amplified with PCR and sub-cloned into the *Mlu*I-*Xho*I sites of the pGL3-basic vector. All constructs were confirmed by DNA sequencing.

### Clonogenic assay

For the cell colony-formation assay, 2000 cells were plated in wells of 6-well plates and treated concurrently with different types of drugs. Sixteen hours later, the reagents were removed by washing the cells with PBS for 3 times. The cells were cultured for 2 weeks and then fixed with 4% paraformaldehyde and stained with 0.1% crystal violet. Statistical and quantitative methods were referred to previously reported [26].

### Cell proliferation assay

The cell counting kit-8 (CCK8) (Biosharp, China) was used to evaluate cell proliferation according to the manufacturer’s instructions [27].

### Cell death assay

Propidium iodide (PI) staining was used for the assessment of cell death [28]. Cells were seeded in a six-well plate at a number of 1 × 10^5^ per well and cultured for 30 hours. The experimental group was treated with drugs for 12 hours, while the control group was further cultured for 12 hours. After the cell culture medium was removed, cells were rinsed twice with PBS buffer. Subsequently, 500 μl of binding buffer was added, followed by the addition of 5 μl of DAPI staining solution and 10 μl of PI staining solution. The reaction took place at room temperature and was shielded from light for a duration of 10 minutes. Finally, cells were observed using an inverted fluorescence microscope.

### EdU incorporation assay

In Vitro Imaging Kit (RiboBio, Guangzhou, China) for the detection of 5-ethynyl-2′-deoxyuridine (EdU) incorporation was used according to the manufacturer’s protocol. The EdU-labeled cells were imaged with a confocal microscope [29].

### TEM

For ultrastructural analysis of mitochondria, TEM was used. Stably HMGA1-knockdown or control KYSE30 cells were treated with or without 5 μM erastin or 1 μM ferrostatin-1, and fixed with 2.5% glutaraldehyde in PBS (pH 7.4) at 4 °C for 2.5 hours, washed 3 times with PBS, and postfixed in 1% OsO4 for 2 hours at 4 °C. The samples were then dehydrated through an ethanol gradient and subsequently embedded in Spurr’s resin. Ultrathin sections were then collected and stained with either uranyl acetate or lead citrate and examined using the transmission electron microscope [30].

### Reduced glutathione (GSH) and malondialdehyde (MDA) assay

The intracellular GSH was measured with glutathione assay kit (Solarbio, China) [31]. The relative MDA concentration in cell lysates was assessed using a Lipid Peroxidation Assay Kit (Solarbio, China) following the manufacturer’s instructions. Briefly, the MDA in the sample reacted with thiobarbituric acid (TBA) to generate an MDA-TBA adduct. MDA-TBA adducts were quantified colorimetrically (OD = 532 nm) or fluorometrically (Ex/Em = 532/553 nm) [32].

### Intracellular ferrous ions assay

Intracellular ferrous ions levels were assessed using FerroOrange (Dojindo, Japan) [33]. Cells were then washed with HBSS for 3 times, incubated with 1 μM FerroOrange for 30 min at 37 °C, 5% CO_2_ in an incubator. Finally, the fluorescence was detected by an inverted fluorescence microscope.

### Western blot analysis

Whole cell extract (WCE) was obtained using RIPA lysis buffer (Solarbio, China). The protein concentration was measured by the BCA Protein Assay Kit (Solarbio, China). Samples were subsequently resolved in 10 or 12% SDS-PAGE gels. Proteins were transferred to Immuno-Blot PVDF membrane (Merck Millipore, Germany) and the membrane was blocked in 5% BSA in tris buffered saline containing Tween-20. The membrane was incubated with the stated primary antibody followed by secondary peroxidase labeled anti-rabbit or anti-mouse antibody (Jackson ImmunoResearch, USA). The signals were developed using an enhanced chemiluminescent solution (Biosharp, China).

### RNA isolation, reverse-transcription, and real-time quantitative RT-PCR

Total RNAs were extracted with TRIzol according to the manufacturer’s instructions. Total RNAs were reversely transcribed into cDNA using the PrimeScriptTM RT Reagent Kit (Takara Bio Inc., Shiga, Japan). The SYBR® green Premix Ex TaqTM kit (Takara Bio Inc., Shiga, Japan) was used for real-time PCR analysis, which was performed using Roche Light cycler 480 II.

### Co-immunoprecipitation

Freshly isolated cells were resuspended and lysed with RIPA buffer and incubated on ice for 30 minutes. The lysates were centrifuged at 12,000 rpm for 15 min at 4 °C. The supernatants were precleaned with protein A/G beads at 4 °C for 1 h and centrifuged for the removal of beads. Anti-HMGA1 antibody was then added to the cleaned supernatant and incubated at 4 °C for overnight. Then protein A/G beads were added and rotated for 2 h at 4 °C. The beads were washed with immunoprecipitation buffer 6 times at 4 °C and then mixed with 20 μl of 1× loading buffer for western blot [34].

### ChIP assay

KYSE30/shR-Ctrl and KYSE30/shHMGA1 cells were cross-linked with 1% (v/v) formaldehyde in PBS for 10 min at 37 °C. Subsequently, 0.125 M glycine was added to terminate the reaction and cells were lysed on ice using lysis buffer. Chromatin DNA was sonicated to obtain ~700 bp fragments, followed by incubation with anti-ATF4 antibody. ATF4-bound chromatin was precipitated with protein A/G-agarose. After de-crosslinking, qPCR was employed to analyze the precipitated DNA to determine putative ATF4 binding in SLC7A11 promoter regions [35].

### Luciferase reporter assay

KYSE30/shR-Ctrl and KYSE30/shHMGA1 cells were seeded in 96-well plates and incubated until cells reached 70% confluence. Luciferase activity was measured after 48 h according to the manufacturer’s instructions (Promega, USA). The activity of the pGL3-basic SLC7A11 promoter-luciferase reporter was normalized to that of the renilla luciferase reporter, and compared between KYSE30 cells with HMGA1 normal expression or knockdown and those transfected with empty vector [35].

### Immunohistochemistry and immunofluorescence staining

Adjacent tissue and tumor of patients with esophageal cancer, and AKR subcutaneous tumors from mice were collected, fixed, paraffin-embedded, and prepared into paraffin sections of approximately 5 μm thickness. After the sections were dewaxed, hydrated, antigen-retrieval, and blocked, antibodies against HMGA1, SLC7A11, Ki67, or 4-HNE (1:200) were applied for overnight at 4 °C, and secondary antibodies were incubated at room temperature. DAB (3,3’-diaminobenzidine tetrahydrochloride) staining and hematoxylin re-staining were subsequently performed. Quantification of immunoreaction was completed using the immunohistochemistry (IHC) plugin of ImageJ software [36, 37]. Immunoreactivity-scoring system (H Score, range from 0 to 300) was used, and the staining intensity was graded (0, absence; 1, weak; 2, moderate; 3, strong) [38]. The H Score was calculated using the following formula: H Score = intensity of staining x % of stained cells [39].

Immunofluorescence staining was performed at room temperature. The cells were fixed with 4% paraformaldehyde for 30 minutes, then the fixative was discarded and washed 3 times with 0.1% triton X-100 in 1 × PBS for 10 minutes each time. Cells were blocked with 3% BSA in 0.1% triton X-100/PBS for 1 hour. Cells were incubated with primary antibodies diluted in the blocking buffer for overnight. Cells were washed with 0.1% triton X-100/PBS for 3 times with 10 minutes each. Cells were then incubated with secondary antibodies (goat anti-rabbit IgG H&L Alexa Fluor 488 (1:1000, Abcam, ab150077), goat anti-mouse IgG H&L Alexa Fluor 647 (1:1000, Abcam, ab150115)) diluted in the blocking buffer for 2 hours in the dark. Cells were then washed with 0.1% triton X-100/PBS for 3 times with 10 minutes each. Finally, the cells were stained with DAPI dye for 5 minutes and soaked with ultrapure water for 5 minutes. Cells were observed and images were taken with a confocal microscope [40].

### Assay for transposase-accessible chromatin using sequencing (ATAC-seq)

KYSE30 cells and HMGA1 knockdown KYSE30 cells were pelleted and resuspended in 25 μl lysis buffer and pelleted again. The nuclear pellet was resuspended into 25 μl transposition reaction mixture containing Tn5 transposase from a Nextera DNA Sample Prep Kit (Illumina) and incubated at 37 °C for 30 min. Then, the transposase-associated DNA was purified using a MinElute Purification kit (Qiagen). To amplify the library, the DNA was amplified for twelve cycles using a KAPA Real-Time Library amplification kit (KAPA Biosystems) with Nextera indexing primers. The total amplified DNAs was purified using AmPureXP beads. The quantity and size of amplified DNA was examined by TapeStation to confirm that independent samples exhibited similar fragment distributions. The libraries were sequenced using a HiSeq 4000 with paired-end sequencing (Illumina) [41]. Replicates were generated from three independent experiments.

### ATAC-seq analysis

To assess the quality of ATAC-seq data, FastQC tool was used to examine metrics, such as sequencing error rate, sequencing depth, and fragment length distribution. Preprocessing steps on the raw sequencing data including removing low-quality sequences, adapter trimming, and removing duplicate reads were performed. The following steps were used to analyze the ATAC-seq data. Aligned the preprocessed reads to a reference genome using alignment tool (Bowtie2). Used peak calling algorithms (MACS2) to identify open chromatin regions from the aligned sequencing data, which represented regions of accessible DNA. Annotated the identified open chromatin regions with genomic features using tool (ChIPseeker). This allows to determine the association of the regions with genes and potential functional roles. Enrichment analysis was performed to determine the enrichment of specific functions or pathways. Visualized the results of ATAC-seq data using visualization tool (IGV), which shows the distribution of open chromatin regions, peak heights, and other relevant information [42–44].

### Single-cell RNA-seq data analysis

ESCC single-cell RNA sequencing data (GSE188900) were downloaded from GEO database and reanalyzed using the Seurat package to compare HMGA1 expression levels in different cell subtypes in ESCC. Methods for analyzing the single-cell RNA sequencing data were described previously [45–47]. Briefly, vst model was used to identify 2000 high variable genes for subsequent processing. To reduce the systematic and random batch effects among samples, the embedding of harmony was used to cluster the population and reduce dimensions. Fifteen significant PCA components were used for harmony embedding calculation. The UMAP maps were generated from the Seurat formula and RunUMAP, respectively. Each subpopulation was identified by the classical signatures, and top markers were evaluated. Similarly, the heatmap and violin plot were also generated by pheatmap and Vlnplot respectively.

### Statistical analysis

All data presented are representative of 3 or more experiments with similar results. Quantitative data are shown as mean ± SEM. Statistical analyses were conducted using GraphPad Prism 8.0 (GraphPad Software Inc., California, USA). We conducted a one-way ANOVA to compare the means among multiple groups, followed by either Dunnett’s multiple comparisons test for comparing all other groups to the control group, or Tukey’s multiple comparisons test for comparing the means between each pair of groups. Additionally, we utilized a one-sample t-test to compare the means of the groups with hypothetical values. Linear regression was used to examine the correlation between two variables [37, 48]. Statistical significance was determined using the following criteria, *P < 0.05, **P < 0.01, and ***P < 0.001.

## Results

### HMGA1 is upregulated in ESCC

**Fig. 1:**
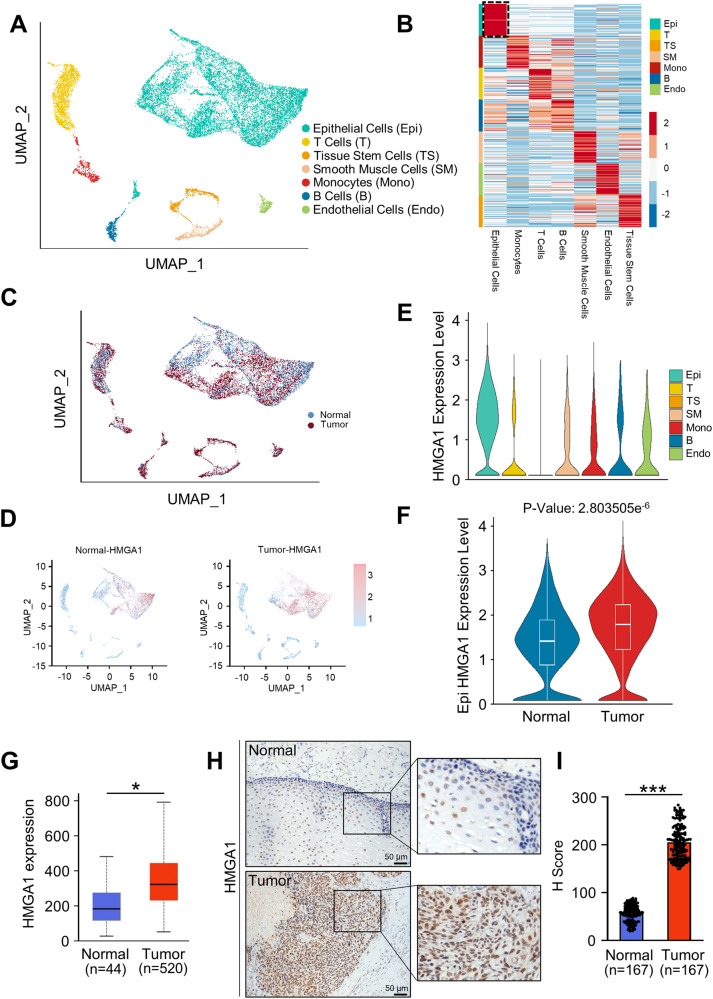
HMGA1 is highly expressed in ESCCs. HMGA1 expression was visualized in ESCC single-cell sequencing (scRNA-seq) data from the GEO database and validated at the histological level. **A** The UMAP map of the single-cell ESCC landscape was colored by cell subtypes. **B** The heatmap showed the expression levels of different cell subtype signatures in scRNA-seq data. **C** The UMAP map showed the distribution of epithelial cells from normal (blue) and tumor (red) in scRNA-seq database. **D** The UMAP map showed expression levels of HMGA1 in various cell subtypes in normal and tumor from scRNA-seq data. **E** Expression levels of HMGA1 in different cell subtypes. **F** Expression levels of HMGA1 in epithelial cells from normal esophagus and tumor in the scRNA-seq data. **G** Differential expression of HMGA1 in esophageal cancers in TCGA database. **H**, **I** Representative IHC staining images of HMGA1 in adjacent tissues and tumors from 167 patients with esophageal cancer. Scale bar: 50 μm. H-scores of HMGA1 expression in tissues were determined. At least 200 cells in each tissue were calculated (***P < 0.001, *n* = 167).

To determine the involvement of HMGA1 in ESCC, we firstly analyzed ESCC single-cell RNA sequencing (scRNA-seq) data derived from the GEO database. Epithelial cells, immune cells, endothelial cells, and other tumor microenvironmental cells were separated, identified, and annotated with classic markers (Fig. 1A). We screened the data for highly differential genes in different cell subtypes and performed a UMAP visualization that separates cell clusters into normal and tumor (Fig. 1B, C). The expression of HMGA1 in epithelial cells of ESCC was positive and significantly higher than that in normal epithelial cells (Fig. 1D–F). Data for HMGA1 expression in ESCCs were also extracted from TCGA database. Consistent with that in the scRNA-seq analysis, HMGA1 was highly expressed in ESCCs (Fig. 1G). To validate our findings, expression of HMGA1 in tumor tissues and corresponding paracancerous tissues from a cohort of 167 patients diagnosed with ESCCs was detected by immunohistochemistry (IHC) assay. It showed that the staining of HMGA1 in ESCCs was much stronger than that in adjacent normal esophageal tissues (Fig. 1H). Semi-quantitation of the IHC staining (H Score) revealed that levels of HMGA1 were significantly increased in ESCCs as compared to those in normal esophageal tissues (Fig. 1I). These results suggest that HMGA1 is upregulated in ESCCs.

### HMGA1 enhances ESCC resistance to cisplatin

**Fig. 2:**
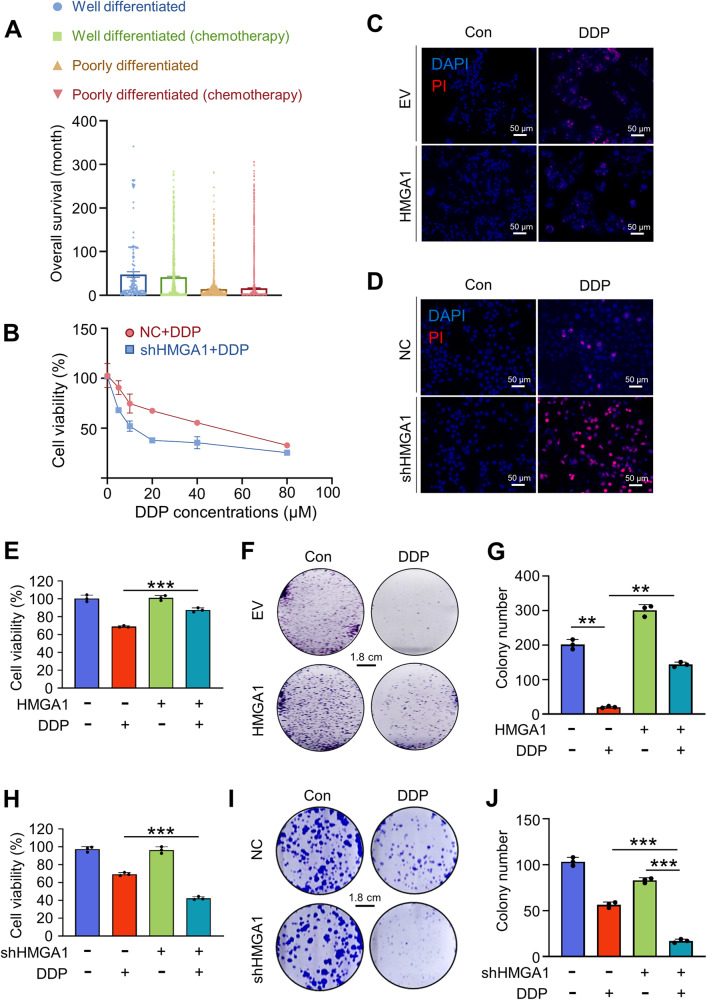
HMGA1 enhances the resistance of ESCCs to cisplatin. **A** Overall survival of esophageal cancer patients categorized by differentiation of tumor and chemotherapy. Data were extracted from the SEER database. **B** Cell viability of control and HMGA1 knockdown KYSE30 cells treated with different concentrations of cisplatin. CCK8 assay was used for the assessment. **C**–**J** Quantification of cell death, cell viability and proliferation in HMGA1-manipulated ESCC cells treated with DDP. **C** and **D**, PI staining was used for detecting cell death in HMGA1 overexpression and knockdown cells treated with 20 μM DPP for 48 h. CCK8 assay (**E**) and colony formation assay (**F**, **G**) to determine cell growth in HMGA1 overexpression KYSE70 cells treated with DDP (20 μM) for 48 h. CCK8 assay (**H**) and colony formation assay (**I**, **j**) to determine cell growth in HMGA1 knockdown KYSE30 cells treated with DDP (20 μM) for 48 h. Colony formation assay was performed using HMGA1 overexpression and knockdown ESCC cells treated with 20 μM DDP for 48 h and cultured for 2 weeks. Scale bar = 1.8 cm. Data represent mean ± SEM. **P < 0.01 and *** *P* < 0.001, *n* = 3.

The effect of chemotherapy on the survival of poorly differentiated and well-differentiated ESCC patients in the SEER database was analyzed. It showed that chemotherapy was unable to improve overall survival in patients with poorly differentiated and well-differentiated ESCCs (Fig. 2A). Overexpression of HMGA1 is known to promote tumor cells proliferation, invasion, and migration [49]. Thus, we detected the impact of HMGA1 expression on the sensitivity of ESCCs to chemotherapy. We assessed HMGA1 expression in various human ESCC cells and in human embryonic kidney 293T (HEK293T) cells (Figure S1A). We then selected ESCC cells with relatively high and low HMGA1 expression to construct ESCC cell lines with HMGA1 knockdown and overexpression (Figure S1B–E), and exposed cells to different concentrations of cisplatin (DDP) (Fig. 2B). Cell death and viability under the treatment of DDP were assessed by PI staining and CCK8 assay respectively. Cell death was decreased correspondingly in HMGA1 overexpressed KYSE70 cells as compared with control KYSE70 cells treated with DDP (Figs. 2C and S2A). Where cell death in HMGA1 knockdown KYSE30 cells was increased compared with control KYSE30 cells treated with DDP (Fig. 2D and S2B). Consistently, KYSE70 cells with HMGA1 overexpression survived better than their wild-type counterparts when challenged with DDP (Fig. 2E). In the colony formation assay, cells with HMGA1 overexpression were less sensitive to DDP and formed more colonies after a 3-week culture as compared with control cells in the presence of DDP (Fig. 2F, G). In contrast, KYSE30 cells with HMGA1 knockdown survived worse than their control partners when challenged with DDP (Fig. 2H). HMGA1-knockdown cells were more susceptible to DDP and formed fewer colonies than DDP-treated control cells (Fig. 2I, J). These results demonstrate that upregulation of HMGA1 reduces DDP-induced cell death and confers ESCC cell resistance to DDP.

### HMGA1 increases ESCC resistance to cisplatin by inhibiting ferroptosis

**Fig. 3:**
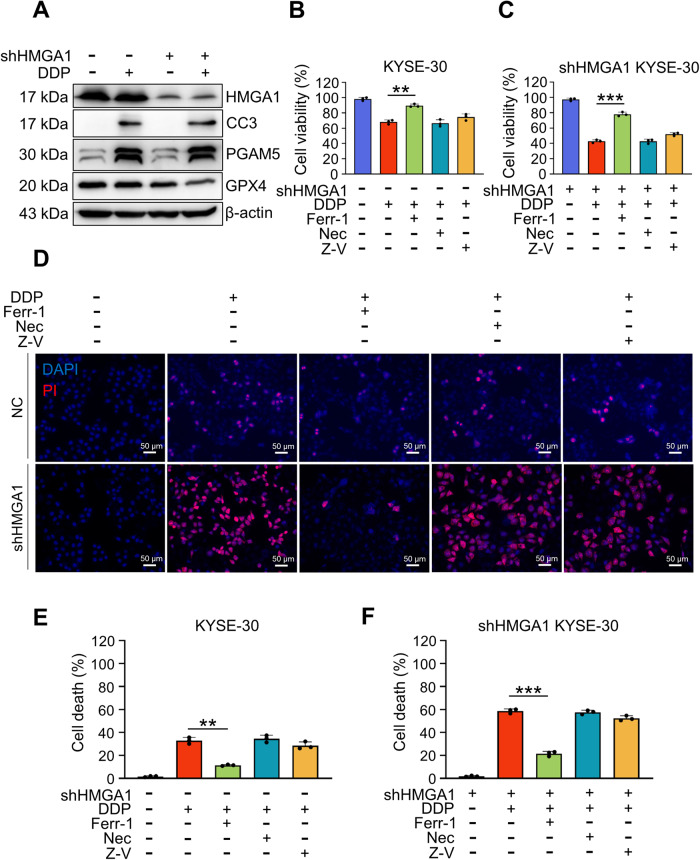
HMGA1 inhibits cisplatin-induced ferroptosis. Inhibitors of ferroptosis, apoptosis, and necrosis were used to treat HMGA1 knockdown KYSE30 cells. **A** HMGA1-manipulated KYSE30 cells were treated with 20 μM DDP for 8 h. After the treatment, whole cell extracts were collected for the western blot analysis. **B**, **C** KYSE30 cells with control and HMGA1 knockdown were treated with 20 μM DDP and 1 μM Ferr-1, 1 μM STS, 20 μM Z-V, or 2 μM Nec for 36 h. CCK8 assays were used for the determination of cell viability. **D**–**F** PI staining was performed to test cell death in HMGA1-manipulated KYSE30 cells treated with 20 μM DDP and 1 μM Ferr-1, 1 μM STS, 20 μM Z-V, or 2 μM Nec for 36 h. Data represent mean ± SEM. **P < .01 and ***P < .001, *n* = 3.

Given that overexpression of HMGA1 reduced DDP-induced cell death in ESCC cells (Fig. 2), we assessed the state of cell death in HMGA1-manipulated cells with DDP treatment. Treatment of DDP led to an increased cleaved caspase-3 and PGAM5 in wild-type KYSE30 cells, suggesting the activation of apoptosis and necroptosis. Interestingly, in HMGA1-depleted KYSE30 cells treated with DDP, there was a substantial reduction in GPX4, a phospholipid hydroperoxidase that protects cells from membrane lipid peroxidation and hence suppresses ferroptosis, whereas the increase in cleaved caspase-3 and PGAM5 was not further enhanced, indicating that ferroptosis may play a role in HMGA1-regulated cell death (Fig. 3A). We used ferrostatin-1 (Ferr-1), necrostatin-1 (Nec), and zVAD-fmk (Z-V) to inhibit ferroptosis, necrosis, and apoptosis in the cells, respectively. It showed that each inhibitor indeed bore a specific inhibitory effect on cell death (Figures S2C, D). To further clarify the impact of HMGA1 expression on cell ferroptosis, we manipulated the expression of HMGA1 in KYSE30 cells and determined cell viability and cell death under the treatment of DDP in the presence or absence of ferroptosis inhibitor, Ferr-1. In control KYSE30 cells, treatment of DDP modestly affected cell viability and cell death (Fig. 3B, E). Depletion of HMGA1 sensitized the cells to the treatment of DDP, leading to a marked reduction in cell viability and a substantial increase in cell death (Fig. 3C, D, and F). More importantly, pretreatment with Ferr-1, but not Nec or z-VAD, blocked the increase in cell death and reversed the reduction in cell viability due to DDP treatment in HMGA1 deficient cells (Fig. 3C, D and F). Taken together, our data suggest that ferroptosis plays an important role in DDP-induced ESCC cell death. Depletion of HMGA1 increases the cytotoxicity of DDP on esophageal cancer cells, in which ferroptosis plays a crucial role.

### Depletion of HMGA1 enhances the sensitivity of ESCC to ferroptosis

**Fig. 4:**
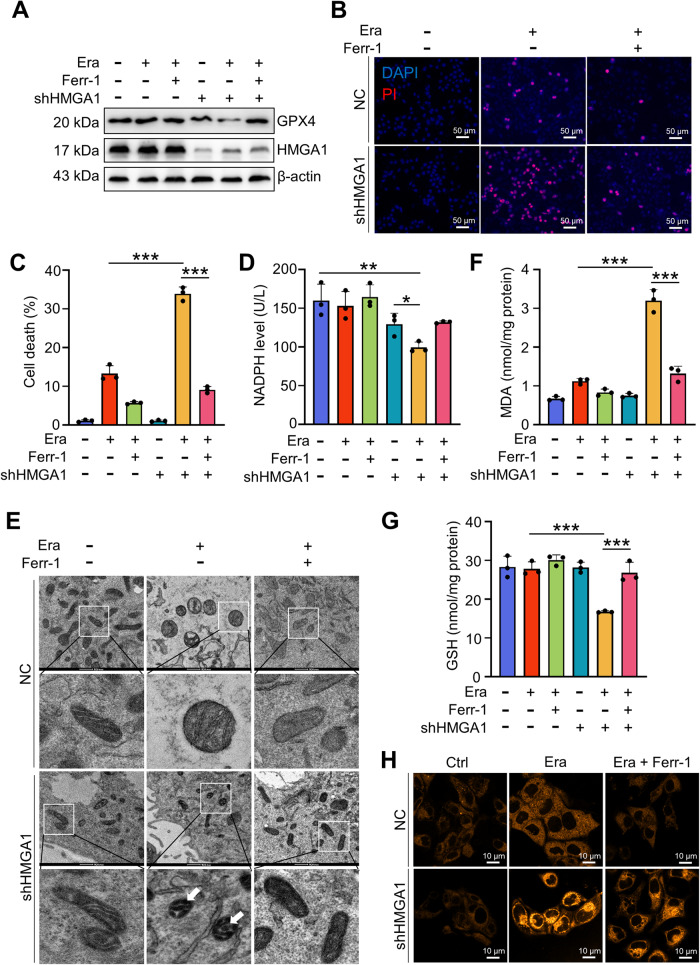
HMGA1 affects ESCC resistance to ferroptosis. The effect of erastin-induced ferroptosis was tested in HMGA1 knockdown KYSE30 cells. **A** KYSE30 control or HMGA1 knockdown cells were treated with 5 μM erastin and / or 1 μM Ferr-1 for 8 h. After the treatment, whole cell extracts were collected for the western blot analysis. **B**, **C**, PI staining for determining cell death in control and HMGA1 knockdown cells treated with erastin (5 μM) for 36 h. **D** NADPH in KYSE30 cells. KYSE30 control or HMGA1 knockdown cells were treated with 5 μM erastin inthe presence or absence of 1 μM Ferr-1 for 12 h. The cells were then collected for the measurement of NADPH by a NADPH assay kit. **E** KYSE30 control or HMGA1 knockdown cells were treated with 5 μM erastin and 1 μM Ferr-1 for 12 h. Cells were then fixed for the analysis by TEM. Scale bars: 500 nm (rows 1 and 3) and 200 nm (rows 2 and 4). **F** MDA assay kit was used to determine the MDA content in control and HMGA1 knockdown KYSE30 cells treated with erastin and/or Ferr-1. **G** GSH assay kit was used to determine the GSH content in control and HMGA1 knockdown KYSE30 cells treated with erastin and/or Ferr-1. **H** FerroOrange (detection probe) was used to determine the content of ferrous ions in control and HMGA1 knockdown KYSE30 cells treated with erastin and/or Ferr-1. Data represent mean ± SEM. *P < 0.05, **P < .01, and ***P < 0.001, *n* = 3.

Our previous data revealed that depletion of HMGA1 promoted the induction of ferroptosis (Fig. 3). To validate the role of HMGA1 in the regulation of ferroptosis in ESCC cells, we induced ferroptosis in HMGA1-manipulated ESCC cells with erastin, a small molecule capable of initiating ferroptotic cell death by inhibiting the cystine-glutamate antiporter system Xct^−^ and glutathione synthesis. Treatment of erastin led to a reduction in GPX4 in HMGA1-knockdown KYSE30 cells (Fig. 4A). Erastin induced a substantial cell death in a low dosage (5 μM) in HMGA1-knockdown KYSE30 cells, whereas the same concentration of erastin in HMGA1-intact cells induced much less cell death (Fig. 4B, C). Consistently, accumulation of nicotinamide adenine dinucleotide phosphate (NADPH), an essential electron donor to provide the reducing power for anabolic reactions and redox balance and inhibition for lipid peroxidation in ferroptosis [50], was also reduced with the treatment of 5 μM erastin in HMGA1-knockdown cells, but not in HMGA1-intact cells (Fig. 4D). Application of ferrostatin-1, an inhibitor of ferroptosis, revoked the alterations of cell death, GPX4 expression, and NADPH in HMGA1-knockdown cells treated with erastin (Fig. 4A–D). These results support our notion that HMGA1 depletion sensitizes the cells to the induction of ferroptosis.

Decrease or disappearance of mitochondrial cristae with an elevated membrane density is a typical morphological feature of ferroptosis. HMGA1-knockdown cells, but not HMGA1-intact cells, treated with erastin showed a typical mitochondrial change in ferroptosis, e.g., fewer and smaller mitochondrial cristae, high density of the mitochondrial membranes (Fig. 4E). In addition, lipid peroxide MDA and ferrous ions increased and the reduced glutathione (GSH) decreased in erastin-treated HMGA1 deficient cells, which were reversed by the pretreatment of ferroptosis inhibitor Ferr-1 (Fig. 4F–H). Collectively, our data suggest that knockdown of HMGA1 enhances the sensitivity of ESCC to ferroptosis induction.

### HMGA1 inhibits ferroptosis through upregulating SLC7A11

**Fig. 5:**
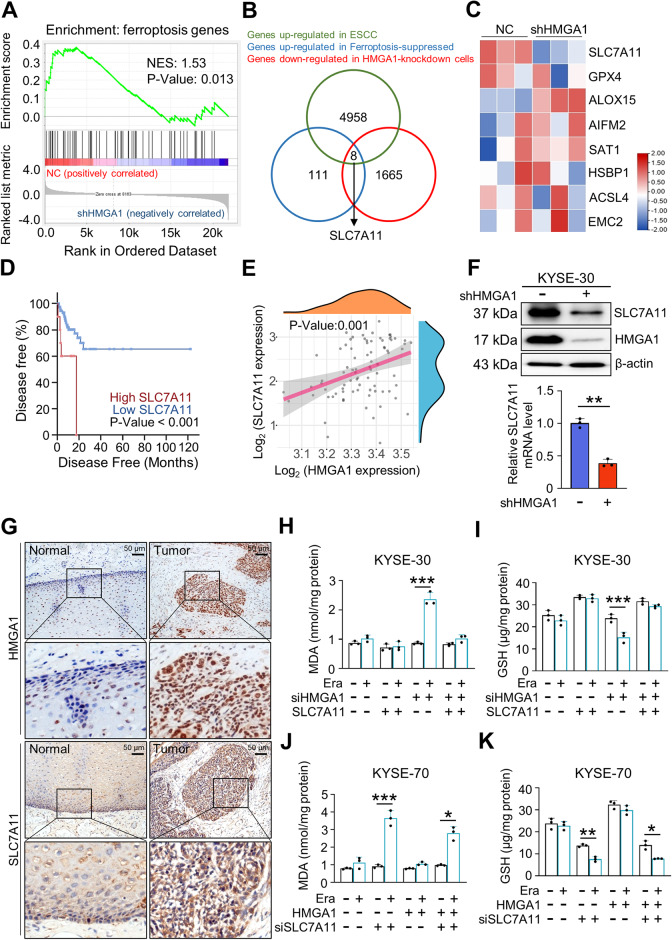
HMGA1 upregulates SLC7A11 to suppress ferroptosis. **A** RNA-seq was performed using KYSE30 control and HMGA1 knockdown cells. Gene Set Enrichment Analysis (GSEA) was used for analyzing RNA-seq data. **B** Venn diagrams showing gene changes in GEO data (GSE23400), FerrDb data, and RNA-seq data in **A**. Overlapped genes in the three cohorts were identified. **C** Heatmap analysis of RNA-seq data in **A** for identifying ferroptosis-associated gene expression in ESCC cells as indicated. **D** Disease-free survival analysis based on SLC7A11 expression in esophageal cancer from TCGA database. The total number of cases is 86, with 10 cases of high expression of SLC7A11 and 76 cases of normal expression of SLC7A11. **E** Spearman’s correlation analysis between HMGA1 and SLC7A11 expression in ESCCs in TCGA. **F** Expression of SLC7A11 protein and mRNA after knockdown of HMGA1 in KYSE30 cells. **G** Representative IHC staining of HMGA1 and SLC7A11 in paired ESCC tissues. The pathological slides are from tumors and adjacent tissues of 40 patients with ESCC. Scale bar: 50 μm. **H**, **J** MDA in KYSE30 cells and KYSE70 cells with the manipulation of HMGA1 and SLC7A11. An MDA assay kit was used for the measurement. **I**, **K**, Determination of GSH content in KYSE30 cells and KYSE70 cells with the manipulation of HMGA1 and SLC7A11 using a GSH assay kit. Data represent mean ± SEM. *P < 0.05, **P < 0.01, and ***P < 0.001, *n* = 3.

To further determine how HMGA1 restricts ferroptosis, we knocked down HMGA1 and performed a transcriptome analysis. Gene-set enrichment analysis (GSEA) showed that a significant expression change in ferroptosis-associated genes occured in HMGA1-depleted cells (Fig. 5A). We compared genes upregulated in ESCC with those suppressed in ferroptosis and those downregulated in HMGA1-knockdown KYSE 30 cells and identified 8 genes shared by above 3 clusters (Fig. 5B, C). *SLC7A11*, coding for the cystine-glutamate antiporter known to suppress ferroptosis, was among the 8 genes and significantly decreased when HMGA1-knockdown (Fig. 5C). Analysis of the TCGA database demonstrated that expression of SLC7A11 portended a poor clinical outcome with markedly short survival of ESCC patients (Fig. 5D). There was a positive correlation between the level of HMGA1 and SLC7A11 in ESCCs (Fig. 5E). Further, knockdown of HMGA1 in KYSE30 cells resulted in a substantial downregulation of SLC7A11 (Fig. 5F), whereas overexpression of HMGA1 increased SLC7A11 expression at both mRNA and protein level (Figure S4A, B).

To assess the functional connection between HMGA1 and SLC7A11 in ESCC patients, we explored the expression of HMGA1 and SLC7A11 in ESCC tissues. Both HMGA1 and SLC7A11 were upregulated in ESCCs as compared with those in normal esophageal tissues. A similar pattern for HMGA1 and SLC7A11 staining was observed in ESCCs (Fig. 5G).

To determine the role of SLC7A11 expression in HMGA1-suppressed ferroptosis, we performed a rescue experiment to overexpress SLC7A11 in HMGA1 compromised KYSE30 cells and silence SLC7A11 in HMGA1 overexpression KYSE70 cells (Figure S4C, D). It was found that there was no significant change in levels of MDA and GSH in HMGA1 intact KYSE30 cells treated with or without erastin. In contrast, HMGA1-knockdown KYSE30 cells treated with erastin bore a much high level of MDA, whereas GSH level was significantly reduced as compared with those in the control-treated cells (Fig. 5H, I). Forced expression of SLC7A11 led to the content of MDA and GSH in HMGA1 depleted cells reaching levels in control or no erastin treatment cells (Fig. 5H, I). Silencing of SLC7A11 led to erastin-induced intracellular MDA accumulation and GSH depletion despite HMGA1 overexpression (Fig. 5J, K). Altogether, our results suggest that HMGA1 inhibits ferroptosis through upregulating SLC7A11. SLC7A11 possibly acts downstream of HMGA1 to inhibit ferroptosis in ESCCs.

### HMGA1 and ATF4 collaboratively regulate the expression of SLC7A11

**Fig. 6:**
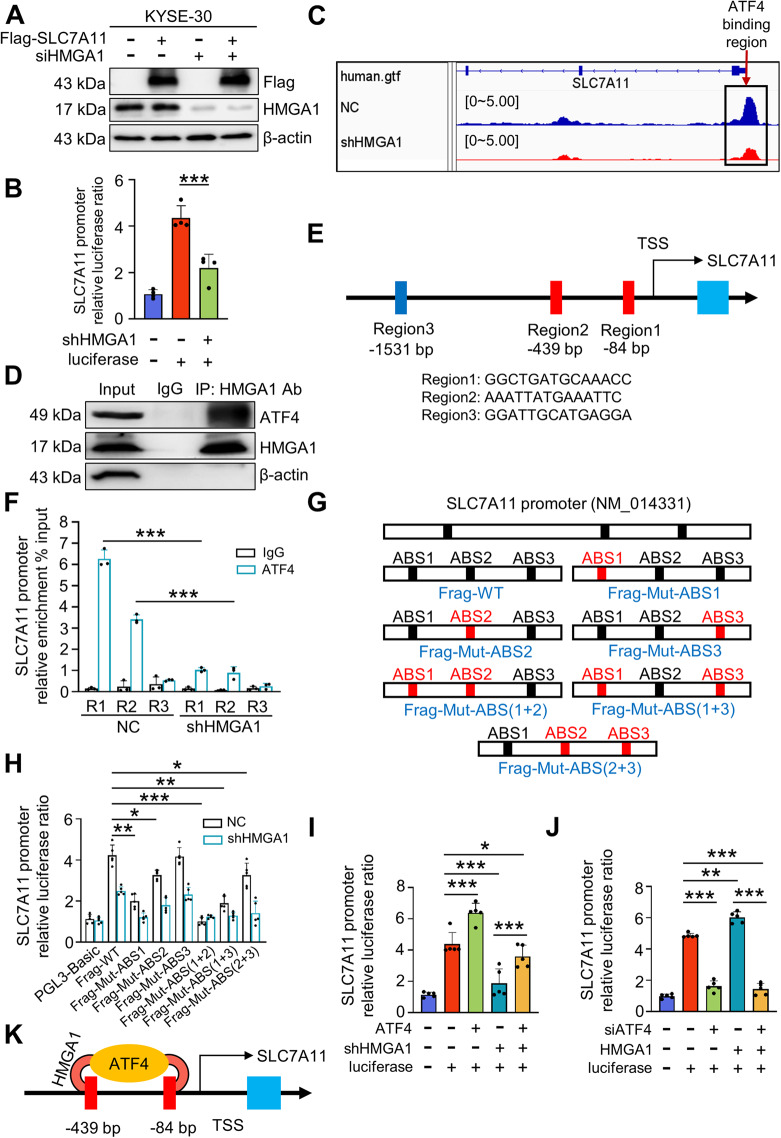
HMGA1 promotes SLC7A11 transcription via ATF4. **A** Western blotting detection of FLAG-SLC7A11 in siHMGA1 KYSE30 cells. **B** Luciferase reporter assays for SLC7A11 promoter activity were performed in KYSE30 cells following HMGA1 knockdown. **C** Control and HMGA1 knockdown KYSE30 cells were analyzed by ATAC-seq. Shown is the SLC7A11 promoter region with transcription factor binding peaks. Boxed is the ATF4 binding site around the transcriptional start site (TSS). **D** Reciprocal immunoprecipitation for detecting the interaction between HMGA1 and ATF4. KYSE30 cell lysates were immunoprecipitated with either IgG or HMGA1 antibody. Input and precipitated proteins were analyzed by immunoblot with the indicated antibodies. **E** Potential binding regions of ATF4 in the promoter of SLC7A11, denoted as Region1, Region2, Region3, were identified based on the sequencing analysis. **F** ChIP assays of KYSE30 cells using an anti-ATF4 antibody or nonspecific IgG. The binding of ATF4 to the SLC7A11 promoter was decreased when HMGA1 was knockdown in KYSE30 cells. **G** Mutations of ATF4 binding site (ABS) sequences in the SLC7A11 promoter. **H** Luciferase reporter assays for the mutant ABS-transduced KYSE30 cells following HMGA1 knockdown. **I**, **J** Luciferase reporter assays were performed in KYSE30 cells with the manipulation of HMGA1 followed by ATF4 overexpression or silence. **K** Diagram showing that HMGA1 assists ATF4 in binding to Region1 and Region2 on the SLC7A11 promoter. Data represent mean ± SEM. *P < 0.05, **P < 0.01, and ***P < 0.001, *n* = 5.

HMGA1 is a chromatin remodeling protein, which acts as a structure transcription factor, mostly affecting the transcription of target genes. Thus, we determined whether the transcription of SLC7A11 is regulated by HMGA1. Similar levels of SLC7A11 were obtained in cells with or without siRNA-HMGA1 treatment when CMV-driven FLAG-SLC7A11-overexpressing plasmid was transduced to KYSE30 cells (Fig. 6A), indicating that HMGA1 does not affect exogenous CMV promoter-induced SLC7A11 expression, but did affect the transcription of SLC7A11 driven by the endogenous promoter (Fig. 6B). To determine whether HMGA1 directly regulated *SLC7A11* gene expression, we analyzed the transcriptional activity of a *SLC7A11*-promoter-luciferase reporter construct in the presence or absence of HMGA1. Indeed, we observed that the *SLC7A11* promoter was fully responsive to the presence of HMGA1, indicating that HMGA1 affects the transcription of *SLC7A11* (Fig. 6B).

Next, we investigated how HMGA1 affects the transcription of *SLC7A11*. Strikingly, a strong enrichment of ATF4 signals was detected within the promoter regions of *SLC7A11*, which is located at chromosome 4 (https://genome.ucsc.edu/). Depletion of HMGA1 remarkably reduced the chromatin accessibility of *SLC7A11*, in which ATF4 binding to the promoter of *SLC7A11* was substantially reduced in the ATAC-seq (assay for transposase-accessible chromatin using sequencing) assay, indicating that HMGA1 may affect the interaction between ATF4 and *SLC7A11* promoter (Fig. 6C). To validate the role of HMGA1 in ATF4-mediated transcription of *SLC7A11*, we performed a coimmunoprecipitation (co-IP) assay and confirmed the interaction between HMGA1 and ATF4 in KYSE30 cells using the ATF4-specific antibody (Fig. 6D). Further, HMGA1 colocalized with ATF4 in the nucleus using the immunofluorescence (IF) staining, supporting the finding that HMGA1 interacts with ATF4 (Fig. 6D and S5).

Next, we employed an online tool Jaspar to search for putative ATF4-binding sites (ABS) in the genomic sequence adjacent to the transcription start site (TSS) of the *SLC7A11* gene. We identified 3 putative ABS within the promoter area of *SLC7A11* (Fig. 6E). Chromatin immunoprecipitation (ChIP) assay confirmed that HMGA1 promoted ATF4 localization to *SLC7A11* promoter region in KYSE30 cells (Fig. 6F). We further identified that ABS1 (R1, -98 bp to -84 bp upstream of TSS) and ABS2 (R2, -453 bp to -439 bp upstream of TSS) were critical for the activation of *SLC7A11* transcription by HMGA1 (Fig. 6F). Depletion of HMGA1 strikingly reduced the binding of ATF4 to the promoter of SLC7A11 in the ChIP assay (Fig. 6F). Next, we mutated each ABS sequence in the promoter area of SLC7A11 individually or simultaneously and measured luciferase activity of SLC7A11 promoter (Fig. 6G). When ABS1 (R1) and ABS2 (R2) were simultaneously mutated, the induction of luciferase activity by HMGA1 was completely abolished (Fig. 6H). In contrast, the luciferase activity was not affected by ABS3 (R3) mutation (Fig. 6H). The results indicate that ATF4 specifically binds to the *SLC7A11* promoter via ABS1 and ABS2 under the condition of normal expression of HMGA1.

To validate the necessarity of ATF4 in HMGA1-promoted transcription of *SLC7A11*, we manipulated the expression of ATF4 and determined the impact of HMGA1 on *SLC7A11* transcription. ATF4 overexpression significantly increased luciferase activity of *SLC7A11* in HMGA1-knockdown KYSE30 cells, reaching that in HMGA1 intact cells (Fig. 6I). In contrast, ATF4 silence abrogated HMGA overexpression-elevated luciferase activity of *SLC7A11* in KYSE30 cells (Fig. 6J). Taken together, these results identify HMGA1 and ATF4 synergistically regulate the expression of *SLC7A11*. HMGA1 promotes the binding of ATF4 to the promoter of *SLC7A11* and enhances the transcription of SLC7A11 (Fig. 6K).

### Depletion of HMGA1 enhances ESCC sensitivity to cisplatin in vivo

**Fig. 7:**
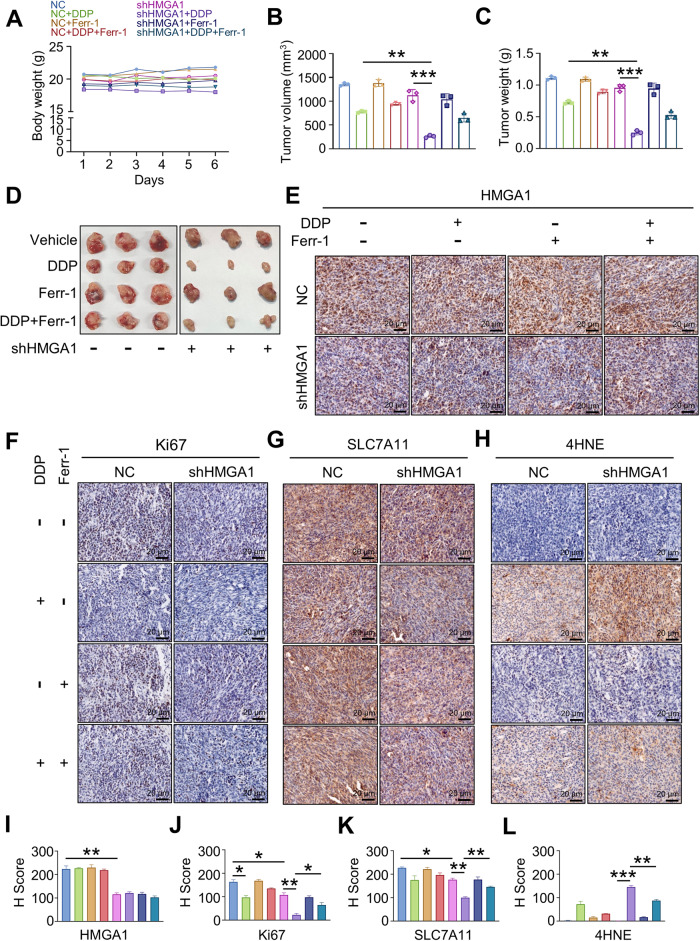
Knockdown of HMGA1 enhances the sensitivity of mouse subcutaneous tumors to cisplatin. Murine esophageal AKR cells were transfected with shRNA of HMGA1 and a stable HMGA1 knockdown cell line was established. One million of the cells were used for the subcutaneous tumor assay. **A** Body weight of mice treated with DDP (5 mg/kg) (*n* = 3). **B**, **C** Tumor volume and weight of subcutaneous allograft tumors developed from esophageal cancer cells with HMGA1 knockdown (*n* = 3). The tumor volumes were calculated according to the formula (L × W^2^)/2 and presented as mean ± SEM. **D** Representative allograft tumors following subcutaneous injection of HMGA1 knockdown AKR cells and treatment with DDP (5 mg/kg) and Ferr-1 (4 mg/kg). **E**–**L**, Representative IHC staining of HMGA1, Ki67, SLC7A11, and 4-HNE in allograft tumor tissues. Scale bar: 20 μm. H-scores of the stainings were calculated. At least 200 cells in each mouse tissue were counted. *P < 0.05, **P < 0.01, and ***P < 0.001, *n* = 3.

Our above results suggest that HMGA1 and ATF4 upregulate *SLC7A11* gene expression (Fig. 6) and thus antagonize ferroptosis, leading to the survival of the cells under the treatment of DDP (Fig. 2C–F and S2A, B). To determine the impact of HMGA1 on ESCC tumorigenesis and the sensitivity of ESCC to DDP, we investigated the functional effect of HMGA1 depletion in combination with DDP on tumors derived from murine ESCC cells (AKR). We subcutaneously injected AKR cells with HMGA1 knockdown into the inguinal of C57BL/6 mice. To demonstrate that inhibition of HMGA1 can sensitize the therapeutic effect of DDP on esophageal cancer tumors, we used a concentration of cisplatin that did not endanger the body weight of mice throughout the treatment period (Fig. 7A). In line with the colony formation assays described above (Fig. 2G–J), HMGA1 deficiency resulted in an increased sensitivity of tumors to DDP treatment in vivo, as reflected by decreased tumor growth as well as by smaller tumor masses at the end point analysis (Fig. 7B–D), which were partially reversed by treatment with the ferroptosis inhibitor ferrostatin-1. In addition, immunohistochemical staining of 4-hydroxynonenal (4-HNE), a highly reactive, cytotoxic aldehyde that is released during the oxidation of w-6-unsaturated fatty acids, confirmed an increase of lipid peroxidation in DDP-treated HMGA1-knockdown tumors, whereas tumor cell proliferation antigen Ki67 as well as ferroptosis suppressor SLC7A11 were significantly decreased (Fig. 7E–L), which was reversed by treatment with the ferroptosis inhibitor ferrostatin-1.

**Fig. 8:**
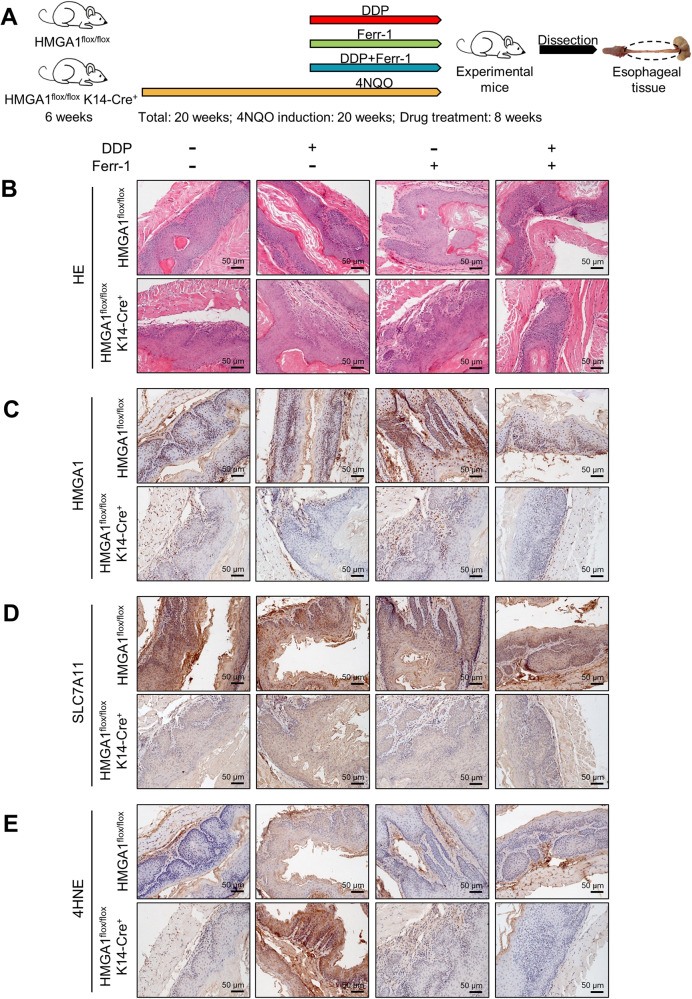
Knockout of HMGA1 enhances the sensitivity of primary ESCCs to cisplatin in mice. **A** A primary ESCC model was established using 4NQO induction in HMGA1^flox/flox^ mice (control) and HMGA1^flox/flox^K14Cre^+^ mice (mice with systemic epithelial cell-specific HMGA1 knockout). **B** Representative HE staining of primary ESCCs in HMGA1^flox/flox^ mice and HMGA1^flox/flox^K14Cre^+^ mice. Scale bar: 50 μm. **C–E** Representative IHC staining of HMGA1, SLC7A11, and 4-HNE in primary ESCCs in HMGA1^flox/flox^ mice and HMGA1^flox/flox^ K14Cre^+^ mice. Scale bar: 50 μm.

To further demonstrate the enhanced therapeutic efficacy of DDP in primary ESCC upon HMGA1 depletion, we utilized genetically engineered mice with systemic epithelial cell-specific HMGA1 knockout to establish an in vivo model of primary ESCC through 4-nitroquinoline-1-oxide (4NQO) induction (Fig. 8A). The histopathological analysis of hematoxylin and eosin (HE) staining displayed that HMGA1 knockout effectively alleviated the formation and size of primary ESCCs and enhanced the therapeutic effect of DDP (Fig. 8B). The IHC staining validated our previous finding in Fig. 5G that HMGA1 and SLC7A11 shared a similar pattern in ESCCs and that depletion of HMGA1 led to a reduced expression of SLC7A11 in primary ESCC tissues (Fig. 8C, D). Furthermore, the IHC staining of 4-HNE confirmed an increased lipid peroxidation in ESCC tissues from HMGA1 knockout treated with DDP (Fig. 8E), supporting our original notion that DDP significantly promotes ferroptosis in HMGA1-deficient ESCC tissues. These results were further validated by the reversal of the therapeutic effect of DDP after co-treatment with the ferroptosis inhibitor Ferr-1, suggesting that ferroptosis plays a critical role in the mediation of HMGA1 depletion-enhanced therapeutic effect of DDP (Fig. 8C–E and S6A–C). Taken together, our studies demonstrate that the genetic ablation of HMGA1 efficiently sensitizes ESCC tumors to DDP therapy by unleashing ferroptosis to promote cell death.

## Discussion

In the process of tumor proliferation and migration/invasion, a constant supply of glucose and glutamine is required for maintaining the malignant phenotypes of cancer. Synthesis and transport of glutamine are highly dependent on the membrane transporter, SLC7A11 [51]. This study demonstrated that inhibition of HMGA1 led to a substantial decrease in the expression of SLC7A11, which serves as an initiative factor for cell ferroptosis [14]. We provided several lines of evidence demonstrating that HMGA1 plays a critical role in maintaining intracellular redox balance and promoting tumor proliferation by cooperating with ATF4 to activate the transcription of SLC7A11. Furthermore, our data suggest that transcription of SLC7A11 is suppressed upon inhibition of HMGA1, hence impairing the function of SLC7A11 in the maintenance of intracellular redox homeostasis. Thus, inhibition of HMGA1 enhances the sensitivity of cancer cells to DDP through upregulating lipid peroxidation-induced ferroptosis. Such functional specification can principally alleviate DDP resistance by damaging mitochondria through redox imbalance causing cell death [52]. But when mitochondrial dysfunction due to redox imbalance leads to insufficient generation of bioenergetics, impaired mitochondria can cause metabolic disorders in tumors, thereby hindering tumor growth [53]. In the current study, we focused on the role of HMGA1 in the suppression of ferroptosis and targeting HMGA1 for sensitizing chemotherapeutic sensitivity. We found the existence of oxidative-reductive imbalance in HMGA1-manipulated cells, indicating a possible role of HMGA1 in cell metabolism, in particular mitochondrial dysfunction. Thus, it is interesting to investigate the impact of HMGA1 on tumor growth or chemotherapeutic sensitivity by affecting tumor cell metabolism in the future.

In conjunction with the findings that HMGA1 suppresses programmed cell death, we speculate that esophageal cancers with elevated expression of HMGA1 will be resistant to chemotherapy. Indeed, in rapidly proliferating esophageal cancer cells, HMGA1 is one of the pivotal factors in protecting cells from chemotherapy damage [54], but the loss of HMGA1 is not the initial step in inducing programmed cell death. In the current study, cell death in HMGA1 knockdown cells after DDP treatment was compared with that in the control cells and showed that HMGA1 knockdown promoted the induction of ferroptosis by DDP. However, when using erastin and STS to specifically induce ferroptosis and apoptosis, we found that HMGA1 knockdown could make cells more sensitive to erastin-induced ferroptosis (Figure S2C, D). In contrast, STS-induced apoptosis was not markedly enhanced due to HMGA1 knockdown (Figure S2C, D). In this regard, we believe that the expression of HMGA1 in ESCC effectively affects the sensitivity of cancer cells to ferroptosis. The regulation of HMGA1 on the ferroptosis pathway is specific (Fig. 3A and S2C, D).

DDP has been reported to induce programmed cell death under oxidative stress by inducing the production of ROS in cells [55]. The role of DDP coincides with the occurrence mechanism of ferroptosis. SLC7A11 works as a gateway to maintain cellular redox balance and to synthesize glutathione, and hence plays a critical inhibitory role in ferroptosis [56]. To ensure the normal functioning of SLC7A11 in maintaining redox homeostasis, the expression and activity of SLC7A11 are subjected to tight regulation by multiple mechanisms [57]. Among them, the regulation of transcription factors and epigenetic regulators is the core mechanism for controling SLC7A11 [58]. The transcriptional regulation of SLC7A11 is highly compatible with the mechanism of action of HMGA1, suggesting that HMGA1 may regulate SLC7A11 at the transcriptional level to achieve cancer cells’ resistance to ferroptosis. In addition, we showed that HMGA1 does not directly function as a transcription factor in regulating the transcription of SLC7A11. ATF4 is necessary for HMGA1-activated transcription of SLC7A11. ATF4 is actively recruited to the promoter region of SLC7A11 by enhanced chromatin accessibility in HMGA1 highly expressed cells. Notably, ATF4 previously was reported to promote the transcription of genes involved in amino acid metabolism and stress responses when it binds to amino acid response elements (AAREs) in gene promoters, thereby enabling cells to cope with amino acid-limiting conditions [59]. SLC7A11, which is responsible for importing cystine into cells for the synthesis of glutathione and for the antioxidant defense, contains AAREs in its promoter, indicating it is a direct target of ATF4 [60]. Future studies are warranted to determine whether intracellular metabolic changes affected by these amino acids play a role in HMGA1-suppressed ferroptosis.

Since surveillance of damaged mitochondria plays a role in maintaining cancer cell viability by blocking the intrinsic ferroptosis pathway [61], the underlying mechanisms could represent intervention targets for cancer treatment. The inhibition of SLC7A11 on the ferroptosis pathway has been assessed in a variety of cancers previously [58]. Since SLC7A11 is also essential for normal cell growth and development [62], directly targeted inhibition of SLC7A11 may cause unexpected side effects. It is an option to target upstream or downstream signaling molecules of SLC7A11 to induce ferroptosis in tumors. Our data showed that both HMGA1 and SLC7A11 were highly expressed in ESCCs whereas a low expression of HMGA1 and SLC7A11 was evidenced in normal esophageal tissues (Fig. 5G). In addition, we demonstrated that HMGA1 directly upregulated the expression of SLC7A11. Thus, it is reasonable to target HMGA1 for suppression of SLC7A11 and for the induction of ferroptosis to enhance chemotherapeutic efficiency. Consistently, we showed that overexpression of HMGA1 in esophageal cancer correlated with patients’ resistance to chemotherapy. Inhibition of HMGA1 was effective in sensitizing esophageal cancer cells to ferroptosis both in vitro and in vivo, providing a proof of concept for targeting HMGA1 in esophageal cancer treatment. Future drug-discovery studies are warranted to identify HMGA1-specific inhibitors, which may expand the therapeutic arsenal for esophageal cancer and other cancers.

### Supplementary information

Figure S1

Figure S2

Figure S3

Figure S4

Figure S5

Figure S6

Supplementary figure legends

Sequence of primers and recombinant DNA, siRNAs

Original Data File

## Data Availability

The authors declare that all the data supporting our findings in the study are available within the paper and its supplementary information files.
