# Supplementary figures and images for: HMGA1 drives chemoresistance in esophageal squamous cell carcinoma by suppressing ferroptosis
Source: Cell Death Dis. 2024 Feb 21;15(2):158. doi: 10.1038/s41419-024-06467-2 (PMC10881472; doi:10.1038/s41419-024-06467-2)

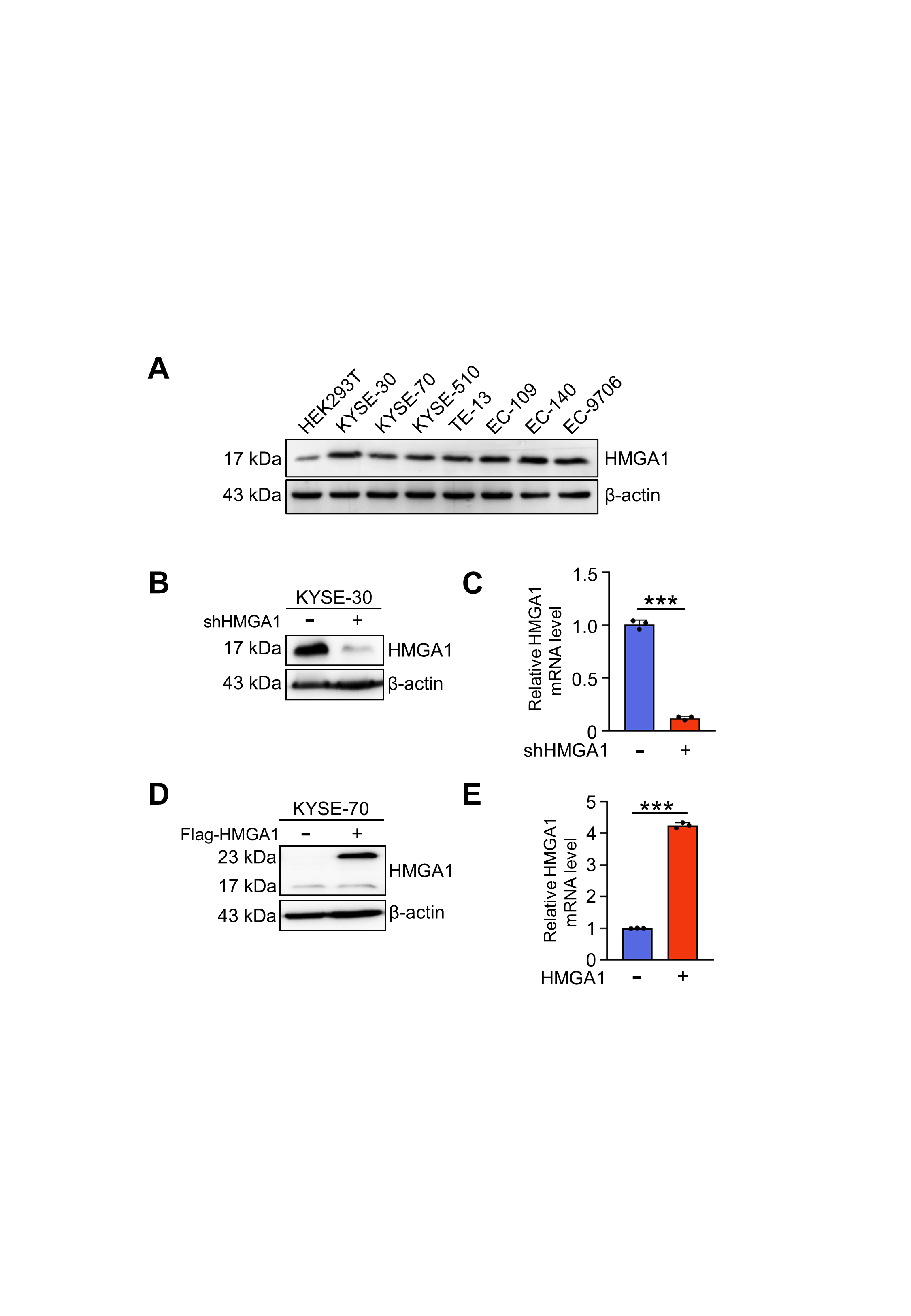

Supplement: Supplementary file 1 — Figure S1 [file 41419_2024_6467_MOESM1_ESM.tif]

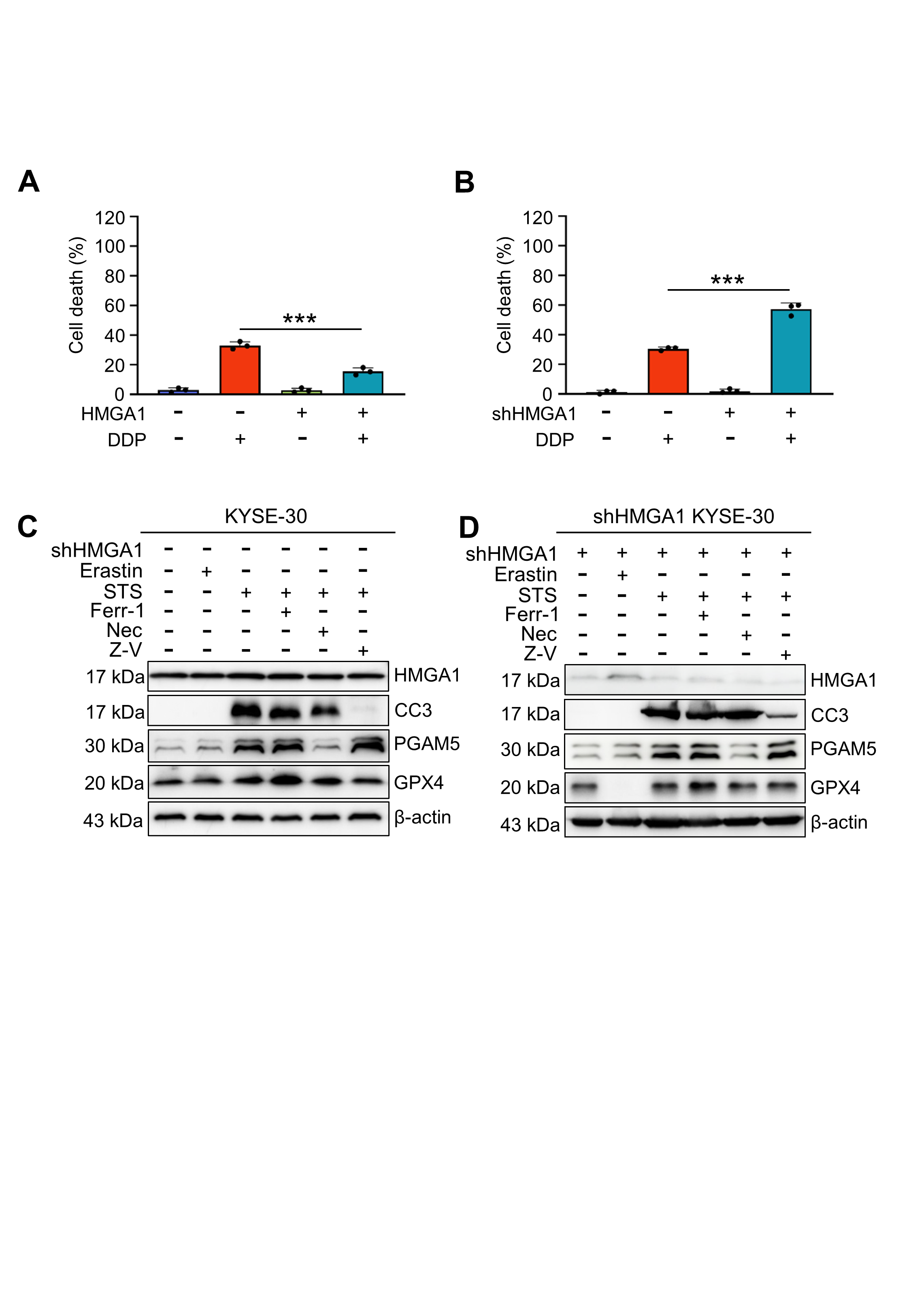

Supplement: Supplementary file 2 — Figure S2 [file 41419_2024_6467_MOESM2_ESM.tif]

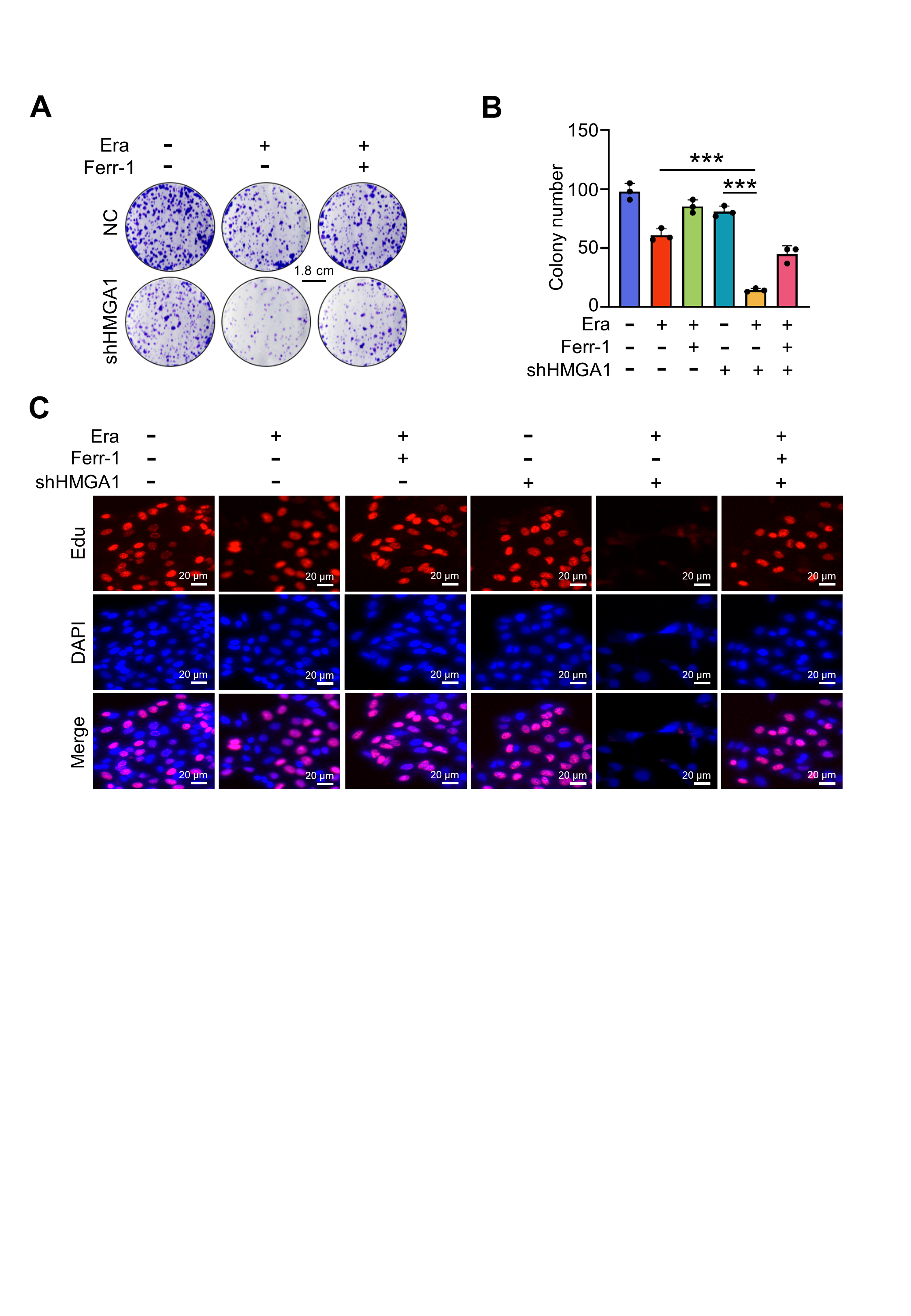

Supplement: Supplementary file 3 — Figure S3 [file 41419_2024_6467_MOESM3_ESM.tif]

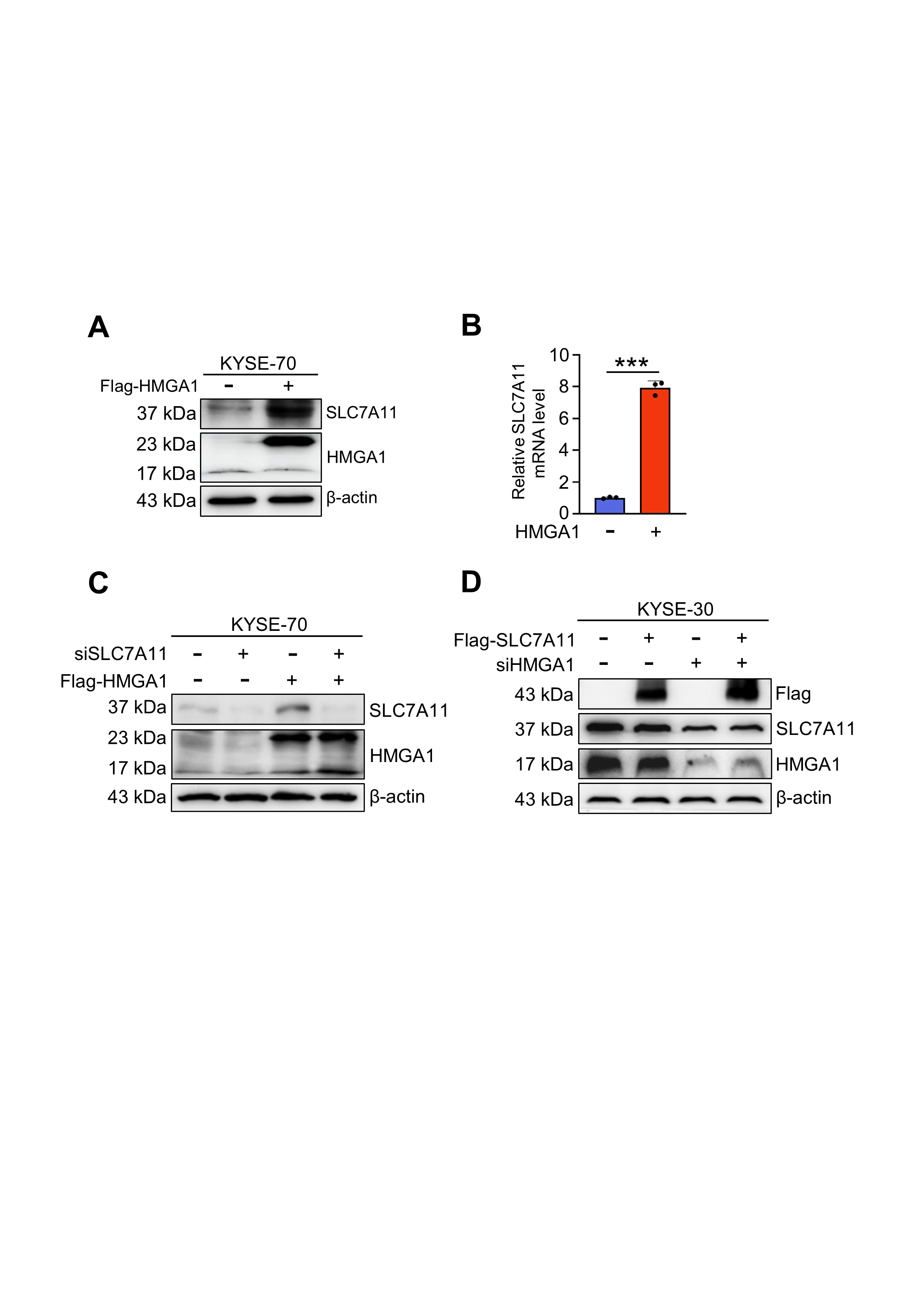

Supplement: Supplementary file 4 — Figure S4 [file 41419_2024_6467_MOESM4_ESM.tif]

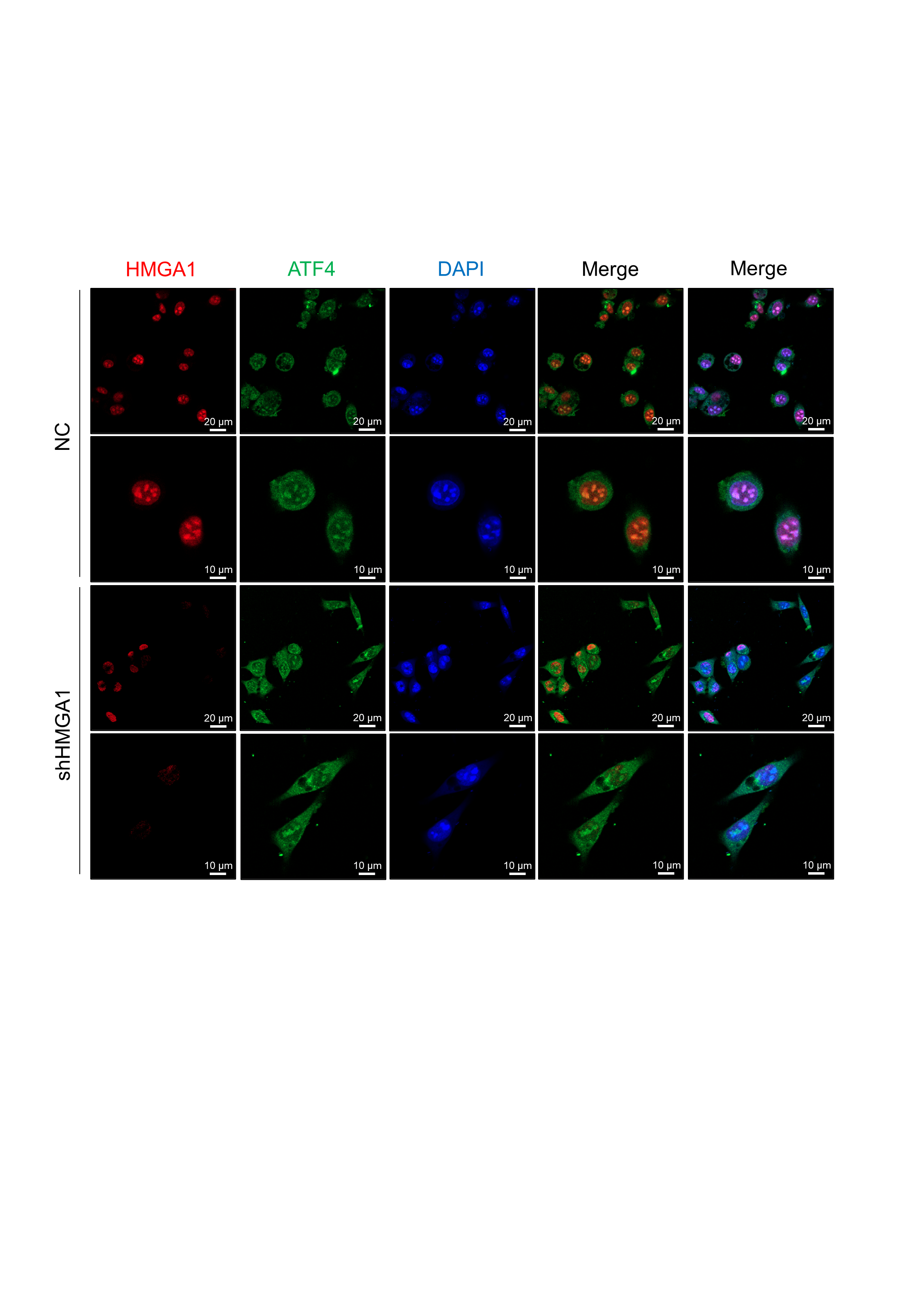

Supplement: Supplementary file 5 — Figure S5 [file 41419_2024_6467_MOESM5_ESM.tif]

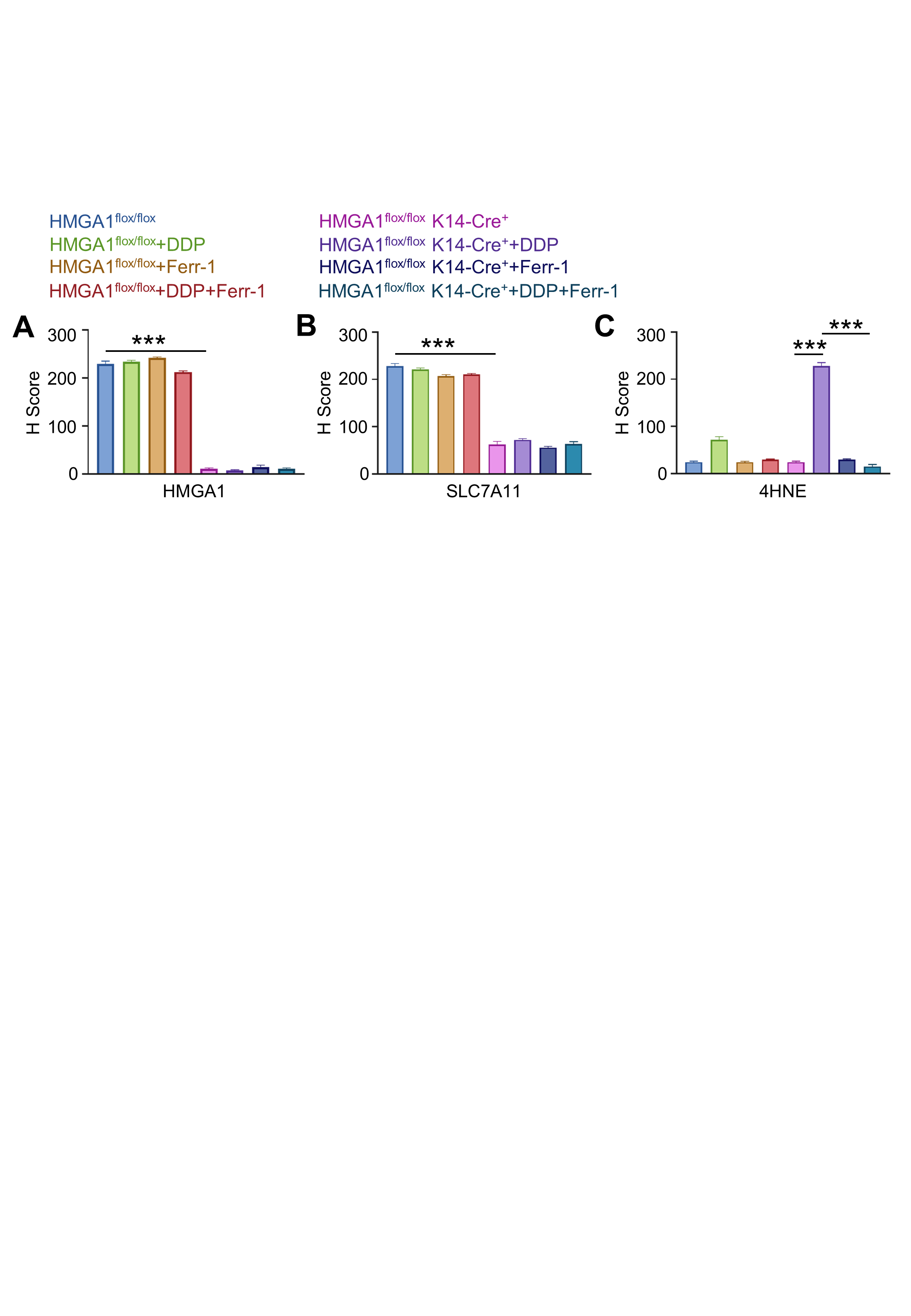

Supplement: Supplementary file 6 — Figure S6 [file 41419_2024_6467_MOESM6_ESM.tif]
