# Supplementary material for: HMGA1 drives chemoresistance in esophageal squamous cell carcinoma by suppressing ferroptosis
Source: Cell Death Dis. 2024 Feb 21;15(2):158. doi: 10.1038/s41419-024-06467-2 (PMC10881472; doi:10.1038/s41419-024-06467-2)
Supplement: Supplementary file 7 — Supplementary figure legends [file 41419_2024_6467_MOESM7_ESM.docx]

**Supplementary Figure Legends**

**Figure S1. Expression of HMGA1 in ESCC cells.**

**a,** Western blot was used for the detection of HMGA1 in human ESCC cell lines (KYSE-30, KYSE-70, KYSE-510, TE-13, EC-109, EC-140, and EC-9706) and in human embryonic kidney 293T cells. **b** and **c,** Validation of HMGA1 knockdown in KYSE-30 cells at protein and mRNA levels. **d** and **e,** Validation of HMGA1 overexpression in KYSE70 cells.

**Figure S2. Cell death in HMGA1-manipulated KYSE30 cells and KYSE70 cells** **treated with DDP.**

**a and b, Quantitation for cell death in** PI staining, which was performed to assess cell death after a 48-hour treatment of DDP (20 μM) in HMGA1 overexpression and knockdown cells. Data represent mean ± SEM. *** P < .001, n = 3 (Accompanied **with Figure 2C and D). c** and **d**, Control and HMGA1 knockdown KYSE30 cells **were treated with 5 μM erastin, 1 μM Ferr-1, 1 μM STS, 20 μM Z-V, and / or 2 μM Nec for 8 h. After treatments, whole cell extracts were collected for the western blot analysis.**

**Figure S3.** **Inhibition of HMGA1 enhances sensitivity to ferroptosis in esophageal cancer cells.**

**a** and **b,** **Control or HMGA1 knockdown KYSE30 cells were treated with 5 μM erastin and 1 μM Ferr-1 for 36 h. Cells were then washed with PBS for 3 times and re-cultured in RPMI1640 for additional 2 weeks for the detection of colony formation. Scale bar: 1.8 cm. c, Cell proliferation was assessed by EdU assay in control and HMGA1 knockdown KYSE30 cells treated with 5 μM Era and / or 1 μM Ferr-1 for 36 hours. Scale bar: 20 μm. Data represent mean ± SEM. *** P < .001, n = 3.**

**Figure S4.** **HMGA1 is positively correlated with SLC7A11 in ESCC cells.**

**a** and **b,** Expression of SLC7A11 at protein and mRNA levels in HMGA1 overexpression KYSE70 cells. Data represent mean ± SEM. *** P < .001, n = 3. **c,** Western blot detection of SLC7A11 and HMGA1 in KYSE70 cells with HMGA1 overexpression and SLC7A11 knockdown. **d,** Western blot detection of SLC7A11 and HMGA1 in KYSE30 cells with HMGA1 knockdown and SLC7A11 overexpression.

**Figure S5. Co-localization of HMGA1 with ATF4 in KYSE30 cells.**

Control and HMGA1 knockdown KYSE30 cells were fixed and subjected to immunofluorescence staining with HMGA1 and ATF4 antibodies. Localization of HMGA1 and ATF4 was observed with a confocal microscope. **Scale bar: 20 μm.**

**Figure S6. Quantitation of HMGA1, SLC7A11, and 4-HNE in ESCCs in HMGA1 knock-in mice.**

**a-c,** H-scores of HMGA1, SLC7A11, and 4-HNE were calculated in IHC stainings of primary ESCCs in **HMGA1^flox/flox^ mice and HMGA1^flox/flox^ K14Cre^+^ mice. At least 200 cells in each mouse tissue were counted. *** P < .001, n = 3 (Accompanied with Figure 8C-E).**
