# Supplementary material for: HMGA1 drives chemoresistance in esophageal squamous cell carcinoma by suppressing ferroptosis
Source: Cell Death Dis. 2024 Feb 21;15(2):158. doi: 10.1038/s41419-024-06467-2 (PMC10881472; doi:10.1038/s41419-024-06467-2)
Supplement: Supplementary file 8 — Sequence of primers and recombinant DNA, siRNAs [file 41419_2024_6467_MOESM8_ESM.docx]

**Supplementary Table. Sequence of primers and recombinant DNA, siRNAs.**

| 1. **Primers used in qPCR analyses** | | |
| --- | --- | --- |
| HMGA1 | Forward primer | CGAGAAAAGGACGGCACTGAC |
|  | Reverse primer | TTGTGGTGGTTTTCCGGGTC |
| SLC7A11 | Forward primer | AAGCACACTCCTCTACCAGC |
|  | Reverse primer | GGATGAACAGTGGCACCTTG |
| GAPDH | Forward primer | AACGGATTTGGTCGTATTGG |
|  | Reverse primer | TTGATTTTGGAGGGATCTCG |
| 1. **Recombinant DNA**  \| **Recombinant DNA** \| **Source** \| **Identifier** \| \| --- \| --- \| --- \| \| V3-HA-FLAG-HMGA1 \| This paper \|  \| \| V3-HA-FLAG-SLC7A11 \| This paper \|  \| \| V3-HA-FLAG-ATF4 \| This paper \|  \| \| V3-HA-FLAG \| This paper \|  \| \| shHMGA1 \| GenePharma \|  \|   **3. siRNAs** | | |
| ATF4 siRNA | Sense | CUCCCAGAAAGUUUAACAATT |
|  | Antisense | UUGUUAAACUUUCUGGGAGTT |
| SLC7A11 siRNA | Sense | GCAGCUACUGCUGUGAUAUTT |
|  | Antisense | AUAUCACAGCAGUAGCUGCTT |
| Negative control siRNA | Sense | UUCUCCGAACGUGUCACGUTT |
|  | Antisense | ACGUGACACGUUCGGAGAATT |
| **4. Primers used in Chip analyses** | | |
| R1 | Forward primer | TGAGTAATGCTGGAGGCTTC |
|  | Reverse primer | TCACACCAACTTACTACAGC |
| R2 | Forward primer | TGAGTAATGCTGGAGGCTTC |
|  | Reverse primer | TCACACCAACTTACTACAGC |
| R3 | Forward primer | GTGAAAGGAGATGTAGGAGG |
|  | Reverse primer | CAGCTACACCAAGTTAAAGT |
| **5. Plasmids used in luciferase assay analyses (SLC7A11 promoter: -2000—+500)** | | |
| PGL3-SCL7A11 promoter (-2000—+500) | This paper |  |
| PGL3-mut (ABS1) | This paper |  |
| PGL3-mut (ABS2) | This paper |  |
| PGL3-mut (ABS3) | This paper |  |
| PGL3-mut (ABS1+2) | This paper |  |
| PGL3-mut (ABS1+3) | This paper |  |
| PGL3-mut (ABS2+3) | This paper |  |
