# Supplementary material for: HMGA1 drives chemoresistance in esophageal squamous cell carcinoma by suppressing ferroptosis
Source: Cell Death Dis. 2024 Feb 21;15(2):158. doi: 10.1038/s41419-024-06467-2 (PMC10881472; doi:10.1038/s41419-024-06467-2)

Fig3A-HMGA1

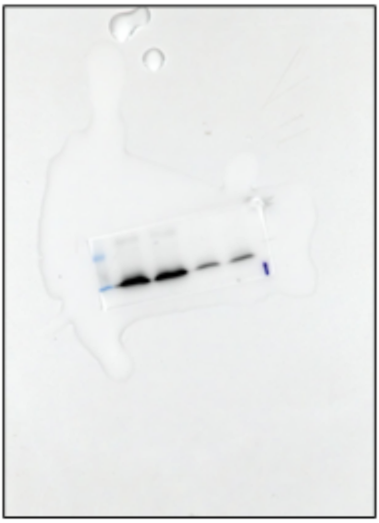

Fig3A-GPX4-Lanes 7-10

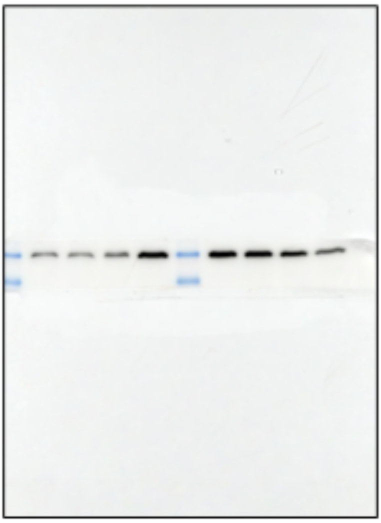

Fig3A-CC3

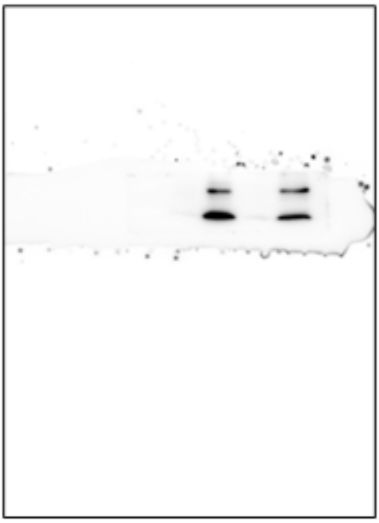

Fig3A-actin

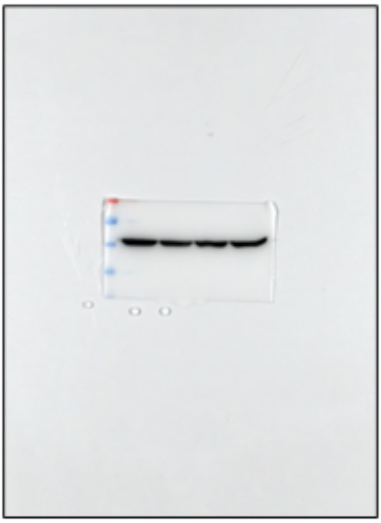

Fig3A-PGAM5

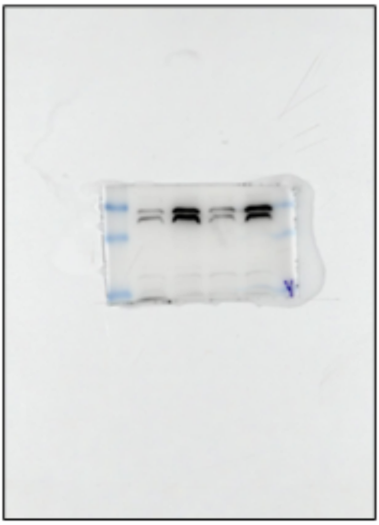

Fig4A-HMGA1

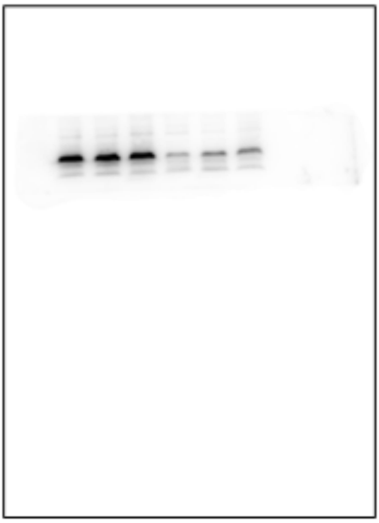

Fig4A-GPX4

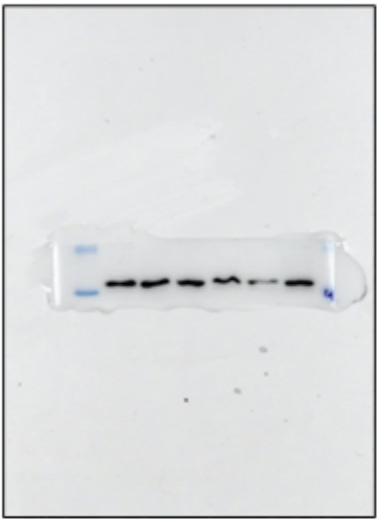

Fig4A-actin

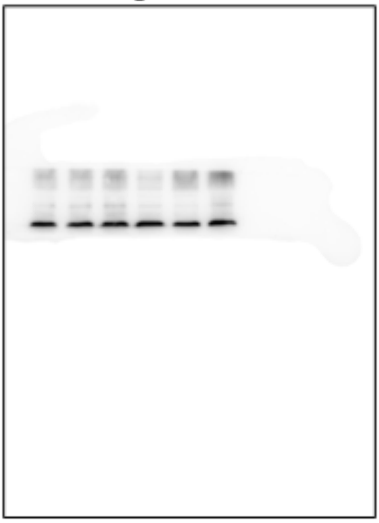

Fig5F-HMGA1-Lanes 2-3

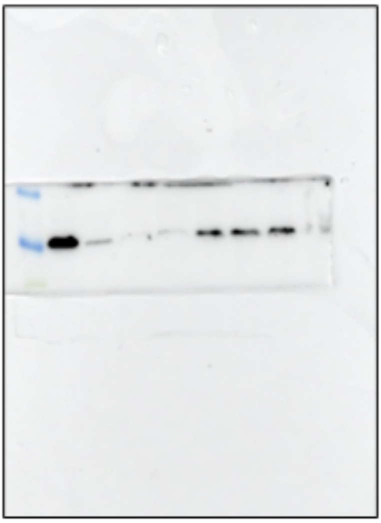

Fig5F-actin-Lanes 2-3

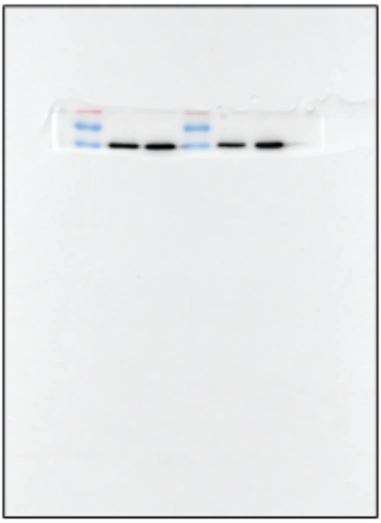

Fig5F-SLC7A11-Lanes 6-7

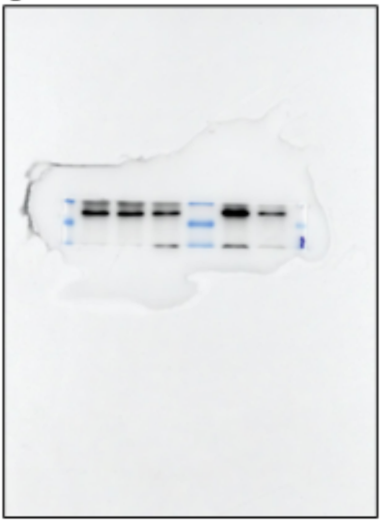

Fig-6A-actin

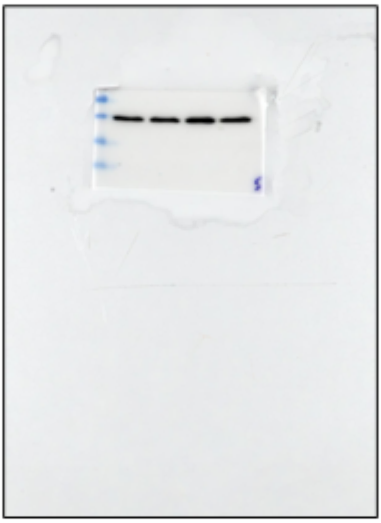

Fig-6A-HMGA1

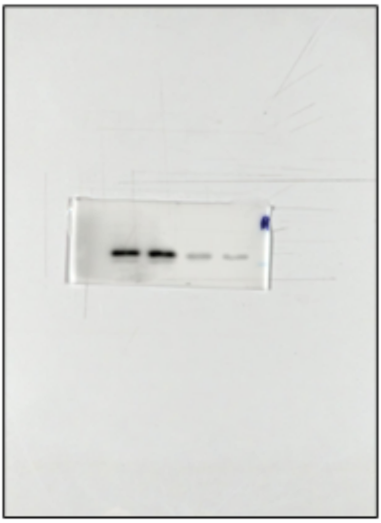

Fig-6A-Flag

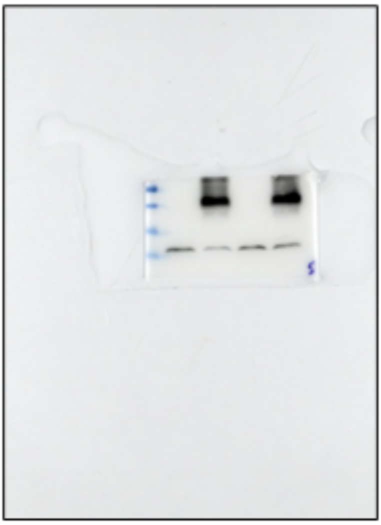

Fig-6D-ATF4

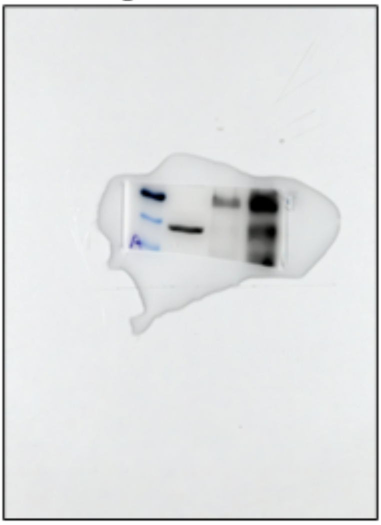

Fig-6D-actin

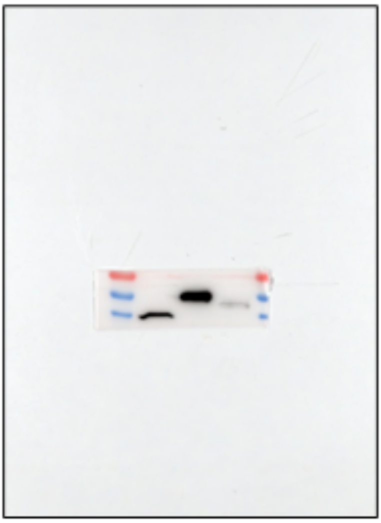

Fig-6D-HMGA1

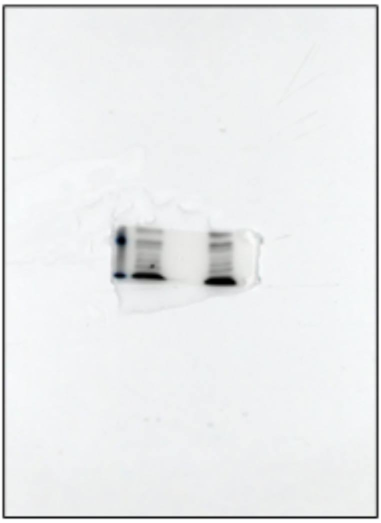

Fig-S1A-actin

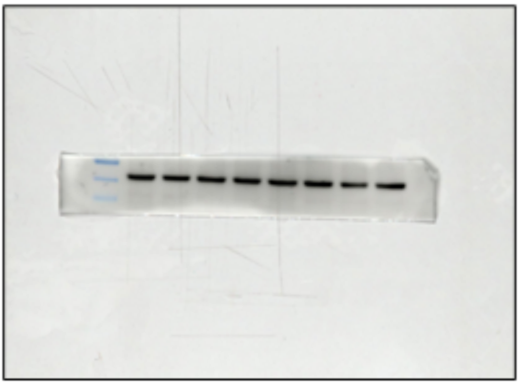

Fig-S1A-HMGA1

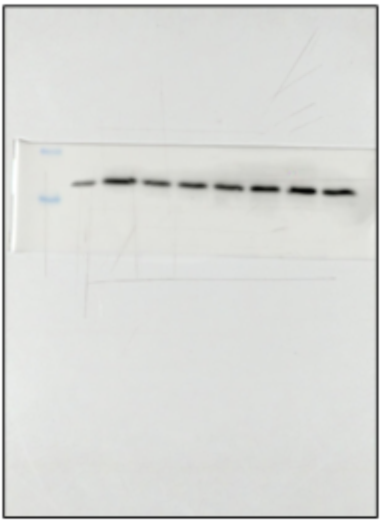

Fig-S1B,D-actin,
S1B-Lanes 7-8,
S1D-Lanes 4-5

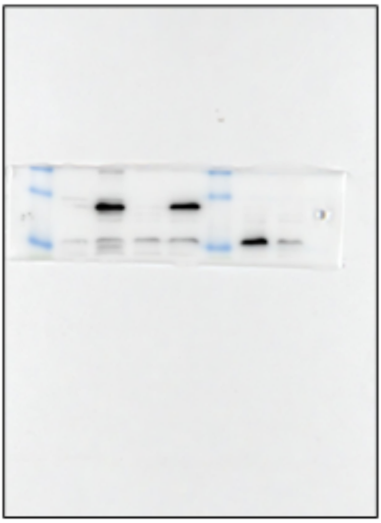

Fig-S1B,D-HMGA1,
S1B-Lanes 7-8,
S1D-Lanes 4-5

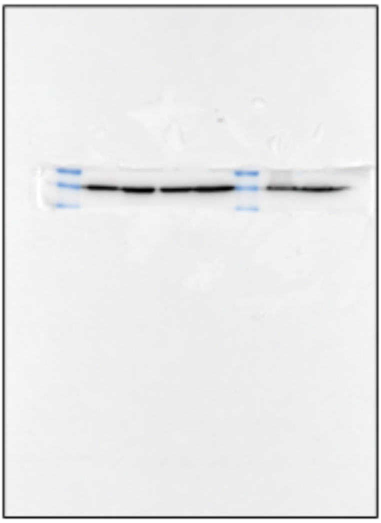

Fig-S2C-HMGA1

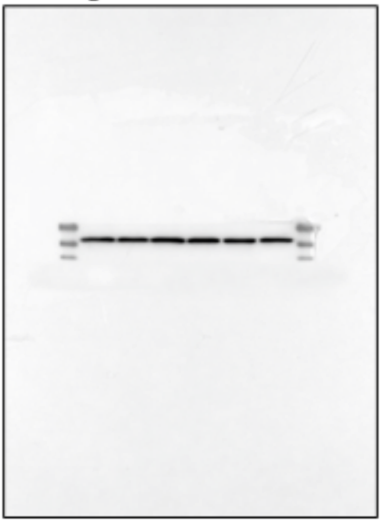

Fig-S2C-GPX4

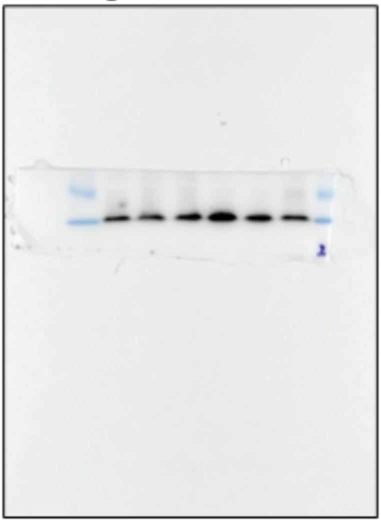

Fig-S2C-CC3

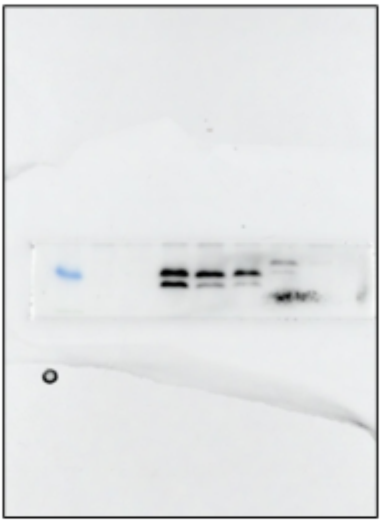

Fig-S2C-actin

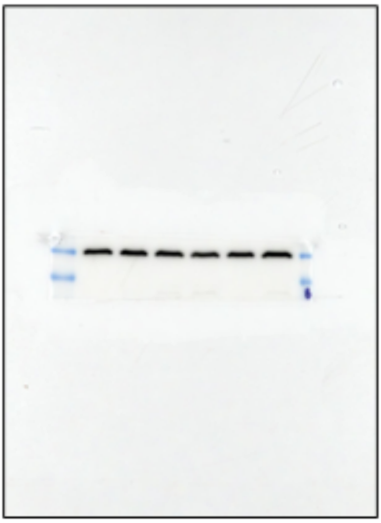

Fig-S2C-PGAM5

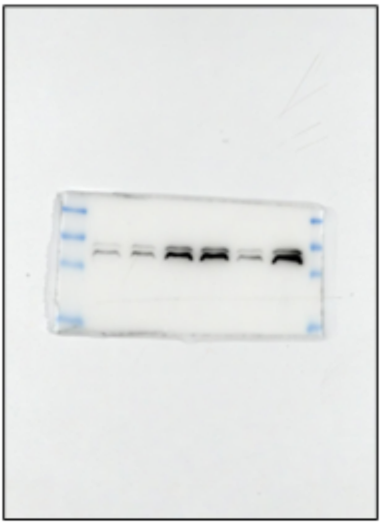

Fig-S2D-HMGA1-Lanes 4-9

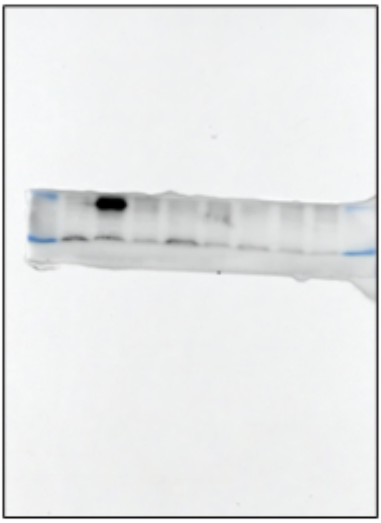

Fig-S2D-GPX4-Lanes 2-7

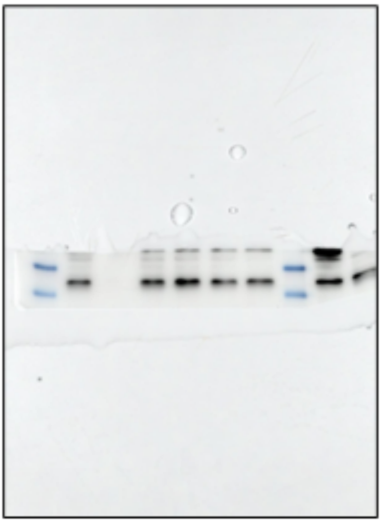

Fig-S2D-CC3

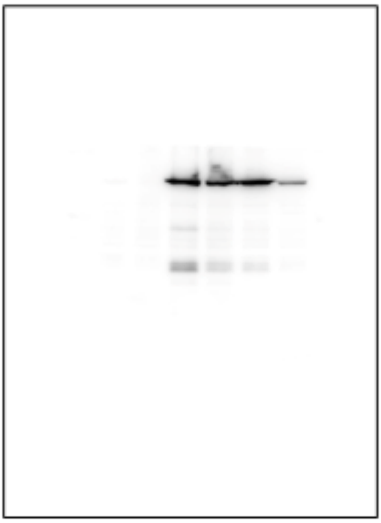

Fig-S2D-actin

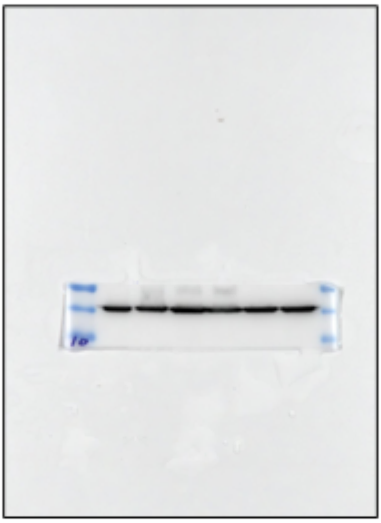

Fig-S2D-PGAM5

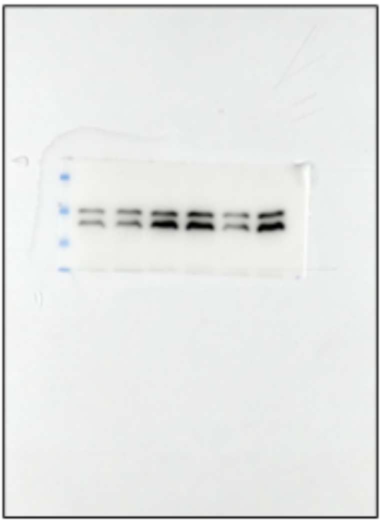

Fig-S4A-HMGA1-Lanes 2-3

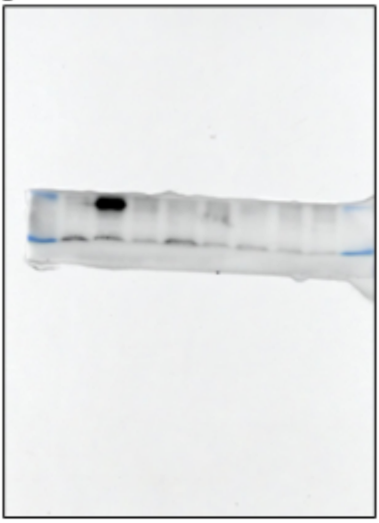

Fig-S4A-actin-Lanes 2-3

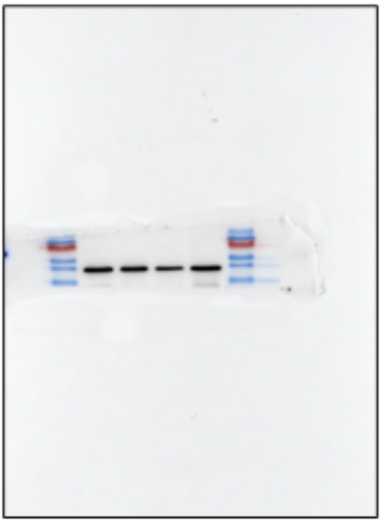

Fig-S4A-SLC7A11

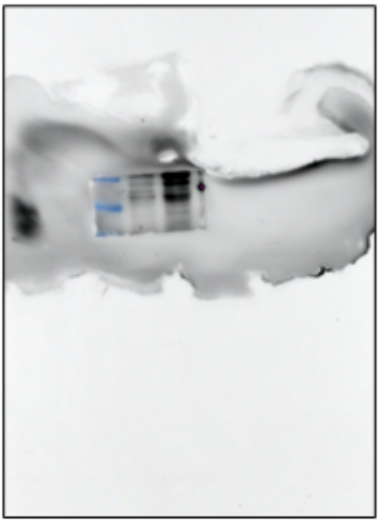

Fig-S4C-HMGA1

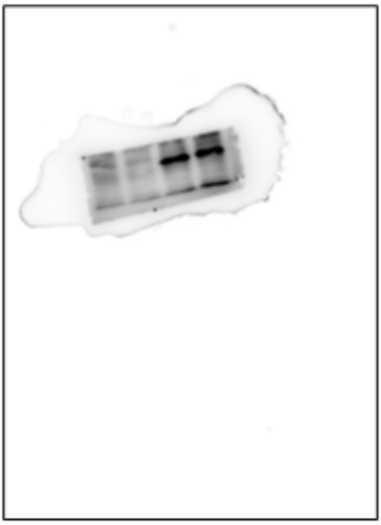

Fig-S4C-SLC7A11-Lanes 2-5

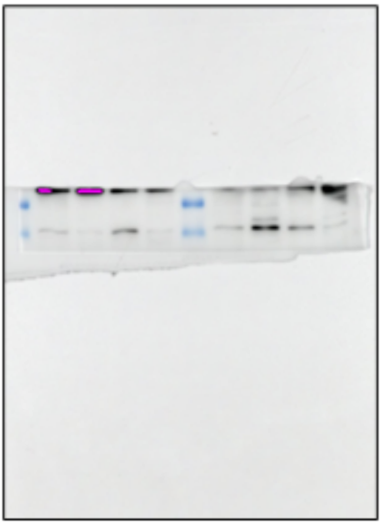

Fig-S4C-actin-The second picture from the top

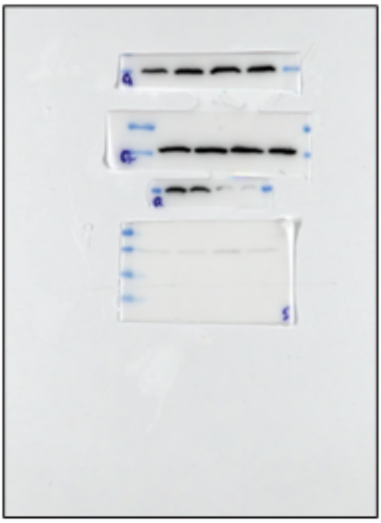

Fig-S4D-HMGA1

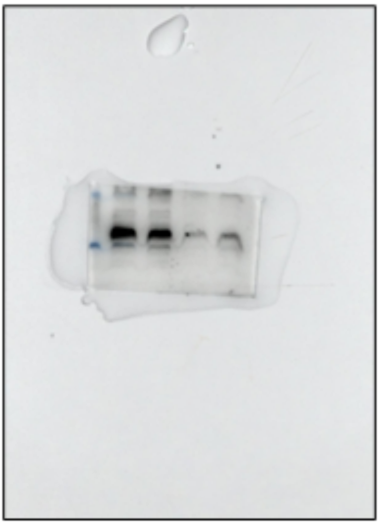

Fig-S4D-Flag

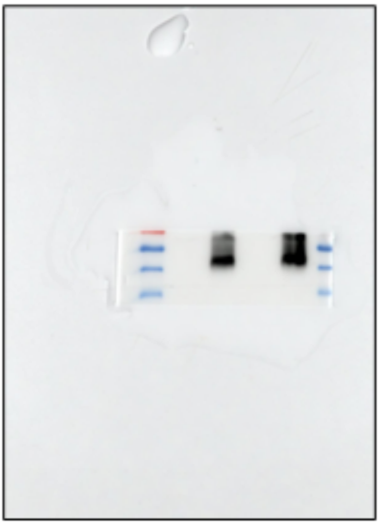

Fig-S4D-actin-Lanes 3-6

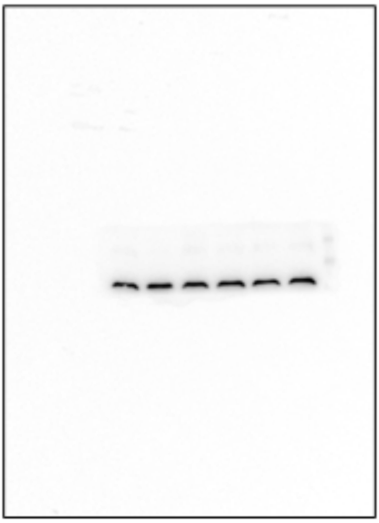

Fig-S4D-SLC7A11

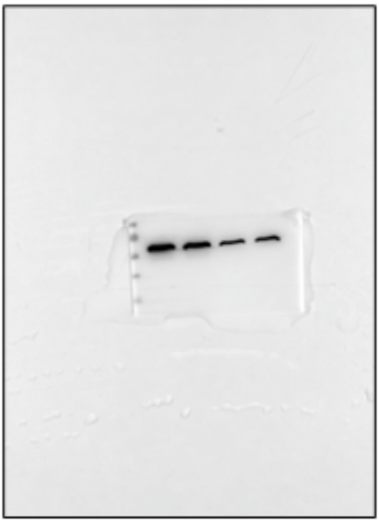

Supplement: Supplementary file 9 — Original Data File [file 41419_2024_6467_MOESM9_ESM.pdf]
